# Rapid TCR:Epitope Ranker (RAPTER): a primary human T cell reactivity screening assay pairing epitope and TCR at single cell resolution

**DOI:** 10.1038/s41598-023-35710-7

**Published:** 2023-05-25

**Authors:** Raquel P. Deering, Lili Blumenberg, Lianjie Li, Ankur Dhanik, Se Jeong, Stephane Pourpe, Hang Song, Lauren Boucher, Shoba Ragunathan, Yanxia Li, Maggie Zhong, Jessica Kuhnert, Christina Adler, Peter Hawkins, Namita T. Gupta, Michael Moore, Min Ni, Johanna Hansen, Yi Wei, Gavin Thurston

**Affiliations:** Regeneron Pharmaceuticals, Inc., 777 Old Saw Mill River Road, Tarrytown, NY USA

**Keywords:** RNA sequencing, Assay systems

## Abstract

Identifying epitopes that T cells respond to is critical for understanding T cell-mediated immunity. Traditional multimer and other single cell assays often require large blood volumes and/or expensive HLA-specific reagents and provide limited phenotypic and functional information. Here, we present the Rapid TCR:Epitope Ranker (RAPTER) assay, a single cell RNA sequencing (scRNA-SEQ) method that uses primary human T cells and antigen presenting cells (APCs) to assess functional T cell reactivity. Using hash-tag oligonucleotide (HTO) coding and T cell activation-induced markers (AIM), RAPTER defines paired epitope specificity and TCR sequence and can include RNA- and protein-level T cell phenotype information. We demonstrate that RAPTER identified specific reactivities to viral and tumor antigens at sensitivities as low as 0.15% of total CD8^+^ T cells, and deconvoluted low-frequency circulating HPV16-specific T cell clones from a cervical cancer patient. The specificities of TCRs identified by RAPTER for MART1, EBV, and influenza epitopes were functionally confirmed in vitro. In summary, RAPTER identifies low-frequency T cell reactivities using primary cells from low blood volumes, and the resulting paired TCR:ligand information can directly enable immunogenic antigen selection from limited patient samples for vaccine epitope inclusion, antigen-specific TCR tracking, and TCR cloning for further therapeutic development.

## Introduction

The identification and functional assessment of antigen-specific T cells from human samples is essential to understand disease pathogenesis, resolve immunogenic antigens, and develop targeted vaccine and TCR-based cellular therapies. The observation that tumors with higher mutation burden (TMB) or viral drivers are more sensitive to immune checkpoint inhibitor (ICI) therapy^1^ has focused much attention toward identifying which tumor antigens elicit functional T cell-mediated anti-tumor responses. Advances in sequencing technologies now permit routine identification of tumor-restricted mutations, and computational algorithms that import bulk whole exome sequence (WES) and RNA sequence (RNA-seq) information from matched tumor and normal tissues can predict which tumor-restricted peptides are likely to be presented on human leukocyte antigen (HLA) alleles^2^. However, the functional relevance of these epitopes must be determined by T cell assays.

There is increasing interest in considering functional T cell reactivity when ranking immunogenic tumor or viral antigens^3^ to better understand which antigens should be therapeutically targeted. Patient functional T cell reactivity tests widely employ traditional, low-throughput assays including enzyme linked immune-spot (ELISpot)^4^ and intracellular cytokine staining (ICS)^5^. ELISPOT and ICS provide functional information by capturing T cells that produce activation-induced T cell cytokines. However, both methods require relatively large numbers of cells to screen reactivities at a high throughput and neither provide TCR sequences.

More recent advances in single cell sequencing technologies have enabled higher-throughput resolution of TCR-ligand interactions. DNA oligonucleotide (oligo)-tagged dextramer technologies permit higher-throughput multimer screening directly on clinical blood samples^6,7^. Notable limitations include the requirement for haplotype matching and dextramer reagent cost. Other sequence-based methods that pair TCR with ligand at a high throughput include yeast display, T-Scan, SABR, cytokine capture, and similar assays^8–13^, although all require engineered expression systems and are not directly applied to clinical samples.

The Multiplexed Identification of T cell Receptor Antigen specificity (MIRA) assay developed by Adaptive Biotechnologies^14^ uses a system of assay well antigen “addressing” to screen T cell reactivities from primary human blood cells. Antigen-specific T cells are identified by upregulation of CD137/4-1BB expression following antigen exposure and sorted for bulk TCR sequencing. While MIRA does not require engineered cells and uses an endogenous T cell activation marker, its method of antigen addressing requires many input cells to capture the low-frequency tumor antigen-specific T cell populations that are accessible in patients’ circulation.

RAPTER was developed to provide a fast, flexible solution for screening candidate T cell antigens. Using an scRNA-SEQ read-out, T cell reactivities of 10–100 s of antigens can be screened in parallel using limited patient blood cells, and paired epitope-TCR information is obtained simultaneously. RAPTER was designed to use autologous human T cells and APCs and requires fewer cells than most other primary T cell assays, which makes it particularly attractive for testing clinical trial samples. Individual antigen reactivities are tracked by labeling T cells with unique hash tag oligonucleotide (HTO)-conjugated antibodies^15^ that correlate to the specific antigens to which the T cells were exposed. To maintain assay sensitivity and decrease the number of cells required for screening, HTO labelling allows all functionally reactive T cells to be pooled, sorted on the same cell surface activation-induced T cell marker (AIM), and undergo scRNA-SEQ. Antigen reactivity is later computationally resolved by HTO demultiplexing (DEMUX). Cellular Indexing of Transcriptomes and Epitopes by Sequencing (CITE-seq) antibody reagents^16^ can also be used in RAPTER to provide T cell surface protein information. Therefore, the RAPTER scRNA-SEQ assay read-out provides paired epitope:TCR information for all detected reactivities, as well as RNA- and protein-level phenotypic T cell Information.

## Results

### RAPTER assay methodology

We sought to develop a functional T cell assay system that uses primary, autologous immune cells to screen 10–100 s of antigen reactivities simultaneously and takes advantage of T cell AIM expression to select antigen-specific cells. RAPTER was developed using either whole PBMC as the source of T cells and APCs, or T cells cultured with autologous monocyte-derived dendritic cells (moDC). All work presented in this paper uses a PBMC culture system as we found this to be a faster and more efficient use of patient blood.

**Figure 1:**
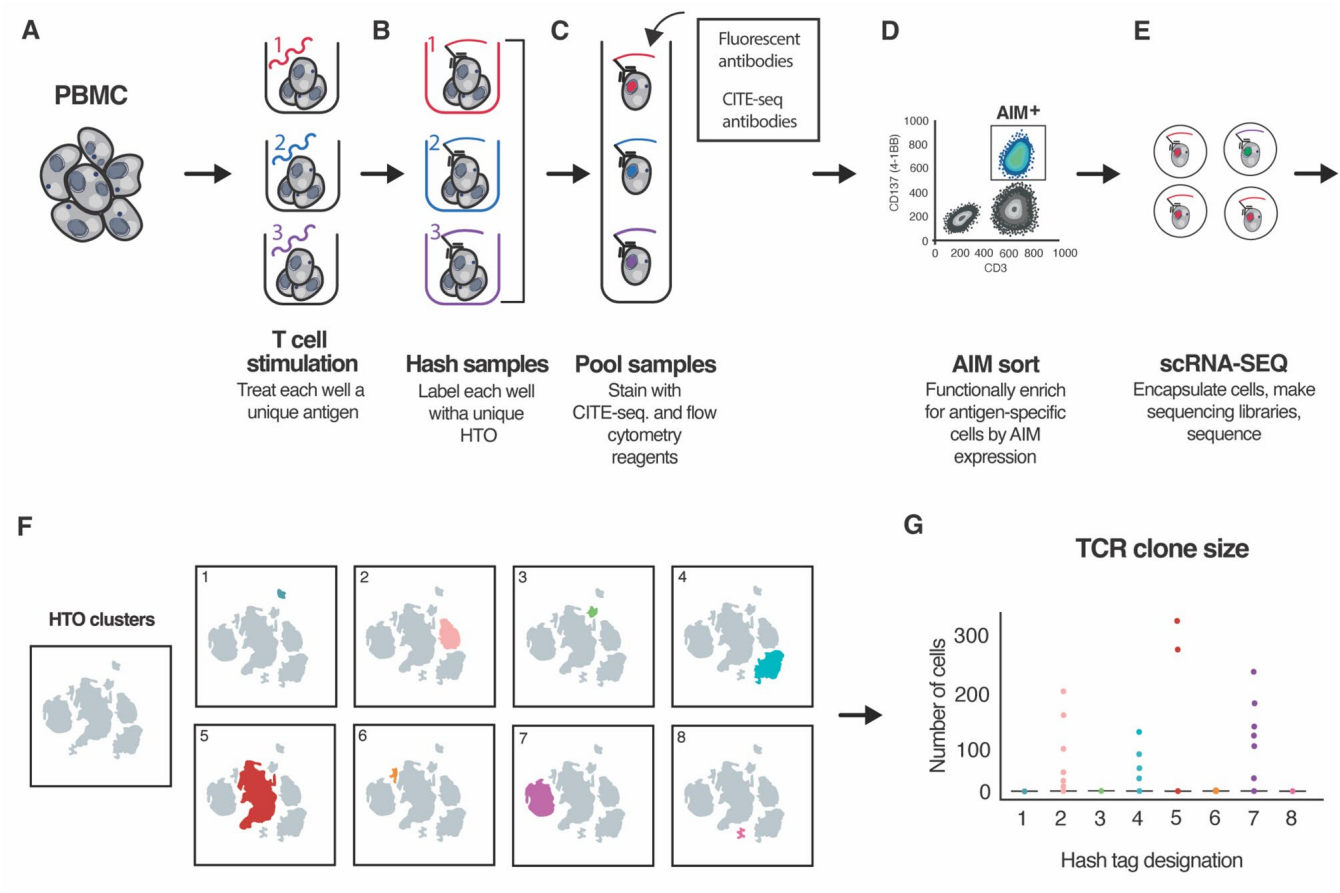
Schematic representation of the Rapid TCR: Epitope Ranker (RAPTER) assay. RAPTER uses hashing reagents and T cell activation-induced markers (AIMs) to select and characterize antigen-specific T cells. (**A**) In this schematic, PBMC are distributed evenly across assay wells. Desired T cell stimuli (protein, peptides, RNA, etc.) are added to individual wells in an arrayed format and cultured with cells for the desired assay time. (**B**) Following stimulation, T cells from each well are labeled with unique hash tag oligonucleotide (HTO)-labeled reagents, e.g., anti-CD2 antibodies, to zip-code the assay well location of all T cells and, hence, their corresponding antigen exposure. (**C**) HTO-labeled samples are pooled and stained with desired flow cytometry and scRNA-SEQ reagents, including fluorescently tagged antibodies and CITE-seq antibodies. (**D**) Antigen-specific T cells are isolated by fluorescence activated cell sorting (FACS) using T cell AIMs for functional enrichment. (**E**) Sorted cells are loaded into the 10X Genomics single cell Chromium partitioner and 5′ sequencing libraries for the transcriptome, HTO, TCR-seq, and, if desired, CITE-seq, and oligo-multimers are generated for HT sequencing. (**F**) Individual HTO assignments are bioinformatically demultiplexed to link individual T cell TCR and phenotype with specific antigen reactivity. (**G**) Antigen-specific TCR sequences are further resolved by evaluating frequency, clone size, and level of CD137/4-1BB expression.

To perform the RAPTER assay, PBMCs are first distributed across assay wells. Either single antigens or antigen pools are then added in an arrayed format (Fig. 1A). Synthetic peptides were used as antigens in this manuscript. After 24 h, T cells from each assay well are stained with an HTO-conjugated antibody (Fig. 1B). While the antibody target for all wells is constant and should target a marker universally expressed by T cells, e.g., anti-CD2, the HTO sequence for each well is unique. Cell target-agnostic lipid-based hashing reagents can also be multiplexed to use in this assay (Supplementary Fig. 1). HTOs are sequenced with the transcriptome using specific primers added to standard scRNA-SEQ protocols. The HTOs mark from which assay well each T cell came, and therefore to which antigen each T cell was exposed.

Once T cells from the individual wells are uniquely labeled with HTO antibodies, all assay wells are pooled (Fig. 1C). Sample pooling provides multiple benefits: consistent staining with surface antibodies for flow cytometry and CITE-seq, sufficient cells for fluorescence activated cell sorting (FACS), reduced use of scRNA-SEQ reagents, and overall reduced technical variation between wells. While a single antigen-specific T cell reactivity might comprise a very low, unsortable population of cells, pooling all wells together provides a larger composite population that permits rapid, efficient sorting. Like others, we designed our strategy to perform scRNA-SEQ, HTO hashing, and oligo-tagged CITE-seq antibody/dextramer reagent staining simultaneously but generated distinct sequencing libraries from each of the three groups to optimize individual library amplification^15^.

The pooled cell population is then sorted using AIM expression as a functional selection marker (Fig. 1D). In at least one assay well, we include a pool of common viral antigens, such as a 35-peptide CMV-EBV-Influenza (CEF) peptide pool, to both serve as a positive control and to ensure a robust, sortable population of cells for FACS and scRNA-SEQ. Sorted AIM^+^ cells are “super loaded” into the 10X Genomics single cell partitioning instrument at an expected yield of 20,000 single cells^17^ (Fig. 1E). Following sequencing, computational HTO demultiplexing is performed to link each T cell with the specific antigen to which it was exposed (Fig. 1F) and HTO-specific TCRs are selected based on clone size and level of 4-1BB expression (Fig. 1G).

### The activation-induced marker CD137/4-1BB identifies antigen-specific CD8^+^ T cells following cognate antigen re-exposure

**Figure 2:**
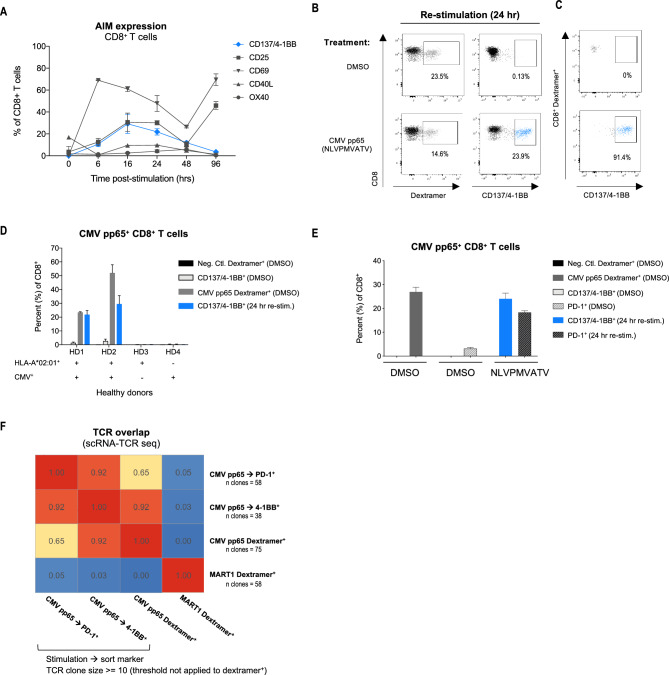
Activation-induced markers are upregulated on antigen-specific T cells following antigen-specific activation. T cells from a CMV serum positive, HLA-A*02:01^+^ healthy donor (HD1) were expanded for 10 days in the presence of CMV pp65 peptide (NLVPMVATV) to enable proof-of-concept tests. (**A**) Pre-expanded T cells were restimulated with cognate CMV pp65 peptide in RAPTER assay culture conditions over a 96-h time course. The percentages of AIM^+^ CD8^+^ T cells was assessed by flow cytometry. (**B**) Pre-expanded T cells were restimulated with DMSO or CMV pp65 peptide (NLVPMVATV) for 24 h. The percentages of CMV pp65 dextramer^+^ and CD137/4-1BB CD8^+^ T cells was assessed by flow cytometry. (**C**) The percentage of CD137/4-1BB^+^ T cells within the dextramer^+^ T cell population post-restimulation was assessed by flow cytometry. (**D**) T cells from HD1 and three additional HDs with varying HLA haplotypes and CMV positivity were expanded for 10 days in the presence of CMV pp65 peptide (NLVPMVATV). The percentages of CMV pp65 dextramer^+^ and CD137/4-1BB CD8^+^ T cells post-restimulation was assessed by flow cytometry. (**E**) Pre-expanded T cells from HD1 were restimulated with DMSO or CMVpp65 peptide for 24 h and the percentages of dextramer^+^, CD137/4-1BB^+^, and PD-1^+^ CD8^+^ T cells were assessed by flow cytometry. (**F**) CMV pp65 and MART1 dextramer^+^ (Dex^+^), and CMV pp65 CD137/4-1BB^+^, and PD-1^+^ CD8^+^ T cells were isolated by FACS then analyzed by scTCR-SEQ. Correlation map shows the overlap between TCR sequences across conditions. All dextramer^+^ TCRs were compared to CD137/4-1BB^+^, and PD-1^+^ TCRs with clone sizes >  = 10 cells.

T cells upregulate a variety of activation markers following antigen-specific TCR signaling, including CD137/4-1BB, CD25, CD69, HLA-DR, CD40L, OX40, and PD-1^18,19^. To initially develop the RAPTER assay, we used HLA class I-restricted viral epitope systems and tested the kinetics of AIM upregulation on CD8^+^ T cells. Primary human T cells from an HLA-A*02:01^+^ healthy donor (HD) with confirmed CMV-specific antibody detection were pre-expanded for 10 days in the presence of CMVpp65_495-503_ (NLVPMVATV) peptide to increase the test population size (Supplementary Fig. 2). Following expansion, the cells were re-stimulated with CMVpp65_495-503_ peptide and the expression of various activation markers, including CD137/4-1BB, CD25, CD69, and PD-1 (Fig. 2A, Supplementary Fig. 3A) was measured at intervals over 96 h. Compared to other AIMs, the percentage of CD8^+^ T cells that upregulated CD137/4-1BB at 24 h post CMVpp65_495-503_ restimulation was comparable in size to the corresponding dextramer^+^ population (23.5% versus 23.9%) (Fig. 2A,B), in alignment with what has been previously reported^20^. Most CD137/4-1BB^+^ T cells co-expressed CD25 and high levels of PD-1 and HLA-DR (Supplementary Fig. 3B), and the kinetics of CD25 expression most closely tracked with CD137/4-1BB expression (Fig. 2A). In the absence of cognate ligand stimulation, all the AIMs tested (HLA-DR, PD-1, CD25, etc.) were expressed to varying degrees at baseline (Fig. 2A, Supplementary Fig. 3A, B) except for CD137/4-1BB (Fig. 2A,B). The extremely low baseline expression of CD137/4-1BB on CD8^+^ T cells makes it an ideal AIM to identify and isolate low-frequency reactivities. AIMs that are co-expressed with CD137/4-1BB^+^ cells, e.g., CD25, can additionally be used to further enrich antigen-specific T cells. Expectedly, the number of detectable CMV pp65 dextramer^+^ T cells was reduced after cognate ligand stimulation, presumably due to TCR internalization, but most T cells that were still detectable by dextramers post-stimulation expressed CD137/4-1BB (Fig. 2B,C). We therefore used mock stimulated (DMSO treated) cells to collect dextramer^+^ cells for all subsequent experiments. Testing additional HDs, we demonstrated that CMV pp65 epitope-specific dextramer^+^ and CD137/4-1BB^+^ T cells were only detected in HDs who were both HLA-A*02:01^+^ and naturally exposed to CMV (Fig. 2D). Together, these experiments show that CD137/4-1BB is a specific and sensitive marker for CD8^+^ T cell epitope reactivity.

### scTCR-SEQ validation that CD137/4-1BB^+^ T cells are enriched for antigen-specific T cells

Having established that CD137/4-1BB is specifically upregulated on CD8^+^ T cells following cognate ligand stimulation, we confirmed that the T cells found in these populations were truly antigen-specific by comparing dextramer^+^ and CD137/4-1BB^+^ clones using single cell TCR sequencing (scTCR-SEQ). We restimulated the CMV pp65^+^ expanded T cells from the HD in Fig. 2A,B with the CMVpp65_495-503_ peptide for 24 h and observed population size agreement across DMSO-treated dextramer^+^ and CD137/4-1BB^+^ T cells by flow cytometry (Fig. 2E). Consistent with previous studies using PD-1 as an AIM to identify antigen-specific T cells in the circulation^21,22^, CD137/4-1BB^+^ T cells also expressed PD-1 (Supplementary Fig. 3B). However, PD-1 was expressed on T cells even prior to re-stimulation with the CMV pp65 peptide (Fig. 2E).

To confirm that the CD137/4-1BB^+^ T cell population was faithfully representing the epitope-specific T cell population, we sorted and performed scRNA- and scTCR-SEQ on CD8^+^ T cells expressing CD137/4-1BB or PD-1 following re-stimulation, as well as CMV pp65 dextramer^+^ T cells and MART1 dextramer^+^ T cells to serve as reference controls (Supplementary Figs. 4 and 5). Expanded TCR clones (clone size >  = 10 cells) overlapped between the dextramer^+^ and CD137/4-1BB^+^ populations more significantly than between dextramer^+^ and PD-1^+^ or PD-1^+^ and CD137/4-1BB^+^ (Fig. 2F). When the overlap among TCR clone sizes > 1 cell was considered, the dextramer^+^ and CD137/4-1BB^+^ samples also correlated significantly (Supplementary Fig. 5C). Expectedly, almost no TCR overlap was observed among the MART1 dextramer^+^ TCRs and CMV pp65-specific TCRs captured by dextramers, PD-1, or CD137/41-BB. These experiments demonstrate that the same epitope-specific TCR clones can be found using CD137/4-1BB and dextramer sorting, but PD-1 was a less specific marker of antigen-specific T cells in these culture conditions.

### RAPTER HTO demultiplexing reproduces the relative frequencies of epitope-specific T cell populations

**Figure 3:**
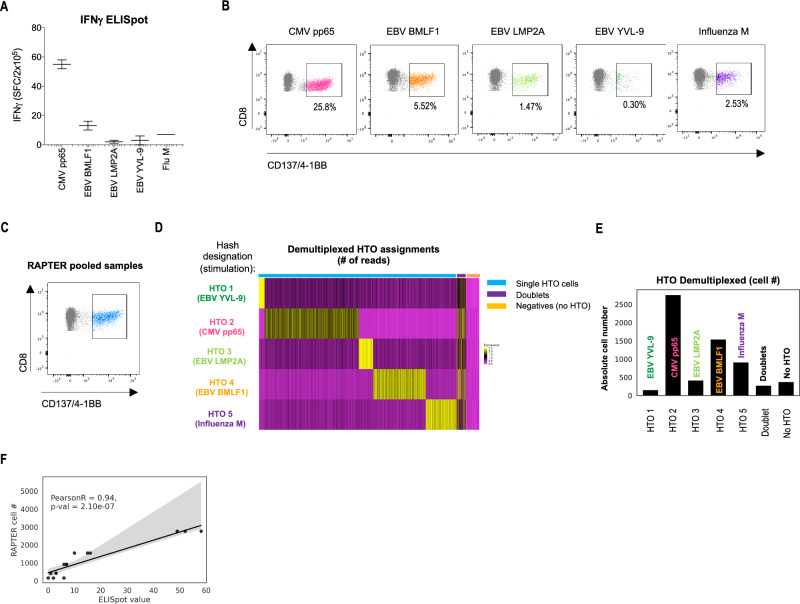
Comparison of ELISpot, functional flow cytometry, and RAPTER. (**A**) Cryopreserved PBMCs from an HLA-A*02:01^+^, CMV^+^ healthy donor (HD1) were tested directly ex vivo in an IFNγ ELISpot assay. The number of IFNγ^+^ spots per 2 × 10^5^ PBMC obtained after stimulation with the 5 indicated HLA-A*02:01-restricted short peptides derived from viral antigens is depicted. (**B**) 20 × 10^6^ PBMC from HD1 were cultured for 7 days with the indicated short peptides to expand the number of epitope-specific T cells. Expanded PBMC were then re-stimulated with peptide for 24 h and the percent of CD8^+^ CD137/4-1BB^+^ T cells for each reactivity was assessed by flow cytometry. (**C**) Expanded PBMC from (**B**) were seeded at 1 × 10^6^ PBMC per well and re-stimulated with the indicated peptides for 24 h and applied to RAPTER. The hashed, pooled CD137/4-1BB FACS gate that was used to collect cells for scRNA-SEQ is indicated. (**D**) HTO assignments for 6,474 captures were bioinformatically demultiplexed from the scRNA-SEQ data. Epitope-specific T cells with single HTO assignments (blue bar) were clustered by their corresponding epitope reactivities, compared to doublets (purple bar) and HTO–negative cells (orange bar). (**E**) Bar plot demonstrating the total number clones assigned to each HTO. (**F**) Correlation plot comparing epitope-specific frequencies between the ELISpot and RAPTER assays.

Having established that CD137/4-1BB^+^ T cells are enriched for epitope-specific TCRs following antigen re-exposure, we tested whether we could recover multiple antigen-specific populations at their respective starting ratios in the RAPTER assay. First, we performed an IFNγ ELISpot assay using cryopreserved PBMC from an HLA-A*02:01^+^ HD to determine the frequencies of reactivities to common viral epitopes (CMVpp65, EBV BMLF1, EBV LMP2A, Influenza M) (Fig. 3A). Given that many patient blood samples are too limiting (fewer than 5 × 10^6^ viable PBMCs) to detect low frequency reactivities directly ex vivo, we also performed a 10-day peptide-specific expansion to increase cell numbers. Then, we assessed the fraction of CD8^+^ T cells that expressed CD137/4-1BB in response to antigen restimulation by flow cytometry (Fig. 3B). The relative abundance of each reactivity determined by the ELISpot experiment performed on unexpanded PBMC was retained during the 10-day expansion (Fig. 3A,B) (Pearson R = 0.99, p-value = 1.17 e-12). Next, we tested the expanded PBMC sample in RAPTER (Fig. 3C, Supplementary Fig. 6). Of the 6,474 total CD137/4-1BB^+^ CD8^+^ T cells sequenced, DEMUX revealed 5 viral antigen-associated populations (Fig. 3D,E) that retained the relative frequencies established by both ELISpot and flow cytometry (Pearson R = 0.94, p-value = 2.1 e-7) (Fig. 3F). We also observed good agreement between scRNA- and CITE-SEQ gene expression for these virus-specific T cells (Supplementary Fig. 7). These experiments demonstrated that the relative frequencies of reactivities were maintained by pre-expansion and RAPTER.

### RAPTER sensitivity test

**Figure 4:**
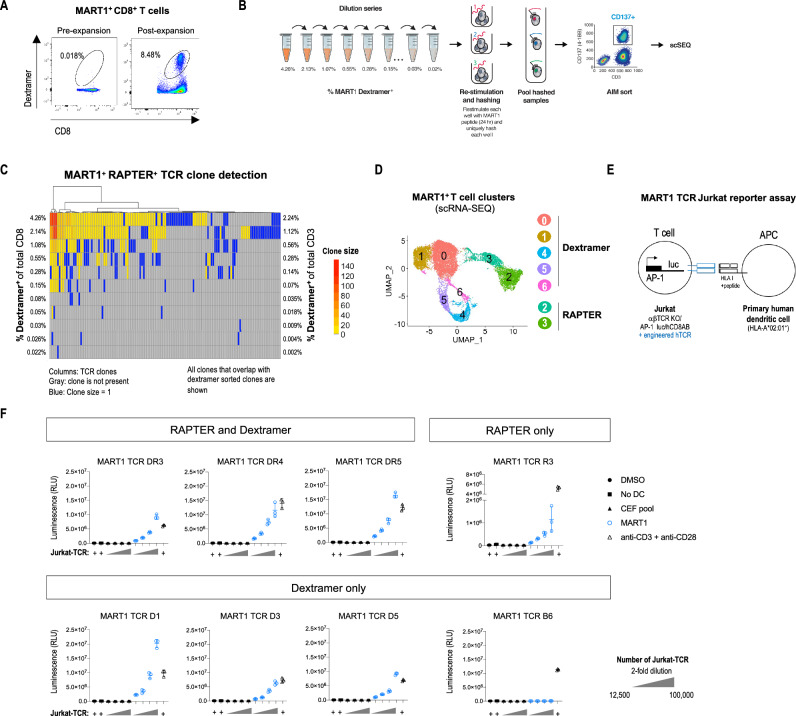
RAPTER limit of detection. PBMCs from an HLA-A*02:01^+^ healthy donor were cultured for 10 days with an HLA-A*02:01-restricted MART1 peptide (ELAGIGILTV) to expand epitope-specific T cells. (**A**) The percent of MART1 dextramer^+^ CD8^+^ T cells pre- and post- expansion was assessed by flow cytometry. (**B**) MART1 peptide-expanded T cells were diluted with unexpanded autologous T cells in a twofold dilution series (theoretical MART1^+^ range: 4.26% to 0.02% of total CD8^+^ T cells) for a total of 50,000 PBMC in each assay well. T cells from each dilution point were re-stimulated for 24 h with the cognate MART1 peptide. Each dilution point was uniquely hashed, then all samples were pooled. CD137/4-1BB^+^ CD8^+^ T cells and MART1 dextramer^+^ CD8^+^ T cells were isolated by FACS for scRNA/TCR-seq. (**C**) HTO demultiplexing was performed to assign CD137/4-1BB^+^, MART1-reactive T cells to specific dilution points. TCR clone sizes range from 1 to 140 cells (Gray: clone is not present. Blue: clone size = 1). All TCR clones that overlap with dextramer sorted clones are shown. Expected percent dextramer^+^ CD8^+^ T cells in each sample is indicated on the left of the TCR clone map. (**D**) Uniform Manifold Approximation and Projection (UMAP) plot of MART1^+^ dextramer-sorted and RAPTER-sorted CD8^+^ T cells. A total of 3554 CD137/4-1BB^+^, MART1-reactive CD8^+^ T cells were captured and comprised 565 unique clones (red) and 9,930 of MART1 dextramer^+^ T cells were captured and comprised 587 unique clones (aqua). MART1^+^ T cells from the dextramer and RAPTER sorts were resolved into 7 distinct phenotypic UMAP clusters based on RNA-seq data. (**E**) Schematic representation of AP-1 firefly luciferase reporter assay. (**F**) Firefly luciferase activity was measured in Jurkat-TCR cultured with dendritic cells treated with DMSO (black circles), pulsed with a CEF peptide pool (black triangles) or the MART1 peptide (blue open circles). Jurkat-TCR cultured alone (black squares) or treated with anti-CD3 and anti-CD28 antibodies (black open triangles) were tested in parallel. MART1 TCRs identified in both the RAPTER and dextramer binding assays (DR3, DR4, DR5), in just the RAPTER assay (R3), in the dextramer binding assay (D1, D3, D5, B6) were tested.

We next sought to determine RAPTER’s quantitative limit of detection. By understanding the lower limits of positive population detection in the assay, the number of simultaneous reactivities that can be queried for a given sample is more accurately approximated. For this proof-of-concept experiment, we focused on an epitope associated with very few antigen-specific T cells at baseline, ELAGIGILTV from the MART1 protein. At baseline, the percent of MART1 dextramer^+^ T cells comprised 0.018% of total CD8^+^ T cells in our HD, but after a 10-day peptide-specific expansion 8.48% of T cells were MART1 dextramer^+^ (Fig. 4A). A dilution series was then performed using autologous non-expanded cells to titrate the antigen-specific target population (Fig. 4B). Cells from each dilution point were re-stimulated with MART1 ELAGIGILTV. After 24 h, the cells were hashed, pooled, sorted using CD137/4-1BB signal, and analyzed by scRNA/TCR-SEQ. Expanded DMSO-stimulated T cells were stained with MART1 dextramers, sorted, and underwent scRNA/TCR-SEQ to provide positive controls with which to compare RAPTER-enriched TCRs (Supplementary Fig. 8). In RAPTER, common MART1 TCR clonotypes are robustly detected (found in >  = 2 cells) starting from a frequency corresponding to 0.15% dextramer^+^ of total CD8^+^ T cells (0.07% dextramer^+^ of total CD3^+^ T cells) (Fig. 4C).

We performed UMAP clustering using scRNA-SEQ data from all MART1 sorted T cells. Unsupervised clustering identified 5 dextramer-sorted and 2 RAPTER-sorted clusters (Fig. 4D, Supplementary Fig. 9). In contrast to the dextramer clusters, both RAPTER populations (clusters 2 and 3) expressed phenotypic markers consistent with T cell activation following antigen restimulation, including high levels of CD137/4-1BB, GZMB, IFNG, CCL4, TNFRSF4 (OX40), and TNFRSF18 (GITR), and low levels of ZNF683 (HOBIT) and SELL (Supplementary Fig. 9). However, RAPTER clusters differed from each other in their functional profiles. Cluster 2 was characterized by high expression of XCL1/2, CD28, IL2RA, and TFRC (CD71) and Cluster 3 by KLRD1, GNLY, and ENTPD1 (CD39), as well as T cell activation markers including PD-1, TIGIT, and KLRD1.

### Functional validation of MART1 TCRs identified by RAPTER

Although most MART1 TCRs were found in both the RAPTER and dextramer-stained samples, some TCRs were identified only by dextramer and, conversely, a few TCRs were present in the RAPTER assay but did not stain with dextramer. Therefore, we cloned TCRs from all categories into a TCR-deficient Jurkat T cell line engineered to expressed luciferase under the control of an AP-1 promoter (Fig. 4E, Supplementary Table 1) and tested their ability to functionally respond to MART1 ELAGIGILTV peptide loaded on moDC derived from HLA-A*02:01^+^ HD PBMCs. All MART1 TCRs that were identified by the RAPTER or by both the RAPTER and dextramer assays, and 3 of the 4 tested MART1 TCRs identified by the dextramer assay, functionally responded to restimulation with the MART1 peptide (Fig. 4F, Supplementary Table 3). Antigen restimulation of one TCR did not lead to T cell activation despite its ability to bind the MART1 dextramer (Supplementary Fig. 10).

### Direct comparison of RAPTER and pooled oligo-tagged dextramer screening

The initial proof-of-concept RAPTER experiments provided us with the necessary technical system to expand to larger epitope screens. To determine the baseline diversity of antigen positive T cells, we used a previously developed pooled oligo-labeled dextramer (dCODE) screening pipeline^23^. We designed a panel of 23 custom dCODE dextramers that included multiple HLA alleles and both virus- and cancer-associated epitopes (Supplementary Table 2). T cells from an HLA-A*02:01^+^, A*29:02^+^, B*35:01^+^, and B*57:01^+^ donor were stained with each dextramer individually (Supplementary Fig. 11) and in a pooled dCODE dextramer screen (Supplementary Fig. 12) to understand the population frequencies and identify antigen-specific TCR sequences. 50 × 10^6^ PBMC were used for these tests, yet many dextramer^+^ T cell populations were present at such low frequencies that they would not be sortable using a single dextramer approach from the available PBMC.

**Figure 5:**
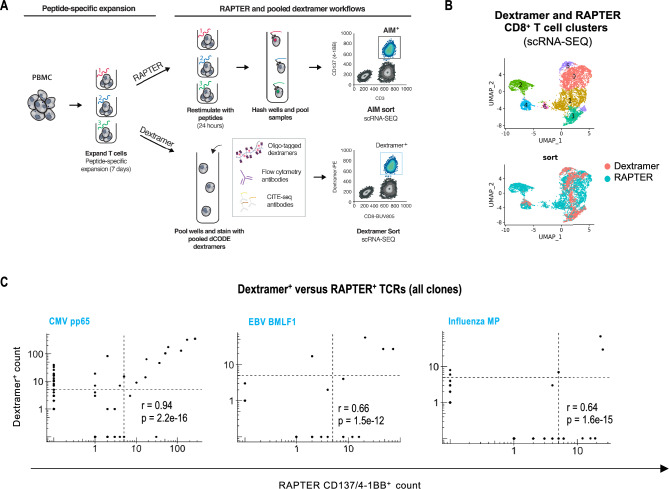
Clonally expanded epitope-specific TCRs are identified in both RAPTER and a pooled oligo-tagged dextramer assays. PBMCs from an HLA-A*02:01^+^, A*29:02^+^, B*35:01^+^, and B*57:01^+^ healthy donor were expanded for 7 days with 22 HLA haplotyped matched and unmatched peptides (Supplementary Table 2) to expand epitope-specific T cells. (**A**) Schematic describing the RAPTER and pooled dextramer workflows that were performed on peptide-expanded T cells. Peptide-expanded T cells were divided and tested in either the RAPTER assay (0.5 × 10^6^ expanded PBMC assay per well; top panel) or a pooled oligo-tagged dCODE dextramer assay with 24 unique pooled dextramers (30 × 10^6^ expanded PBMC; bottom panel). (**B**) UMAP displaying CD8^+^ T cell clusters from the dCODE dextramer and RAPTER assays. (**C**) Correlation plots of paired TCR a/b chain sequence overlap between peptide expanded dextramer^+^ (y-axis) versus peptide expanded RAPTER^+^ (x-axis) CD8^+^ T cells across clone sizes (log10 scale). The dashed lines indicate TCR clone sizes >  = 5 cells.

Having evaluated the effects peptide pre-expansion on TCR repertoire and established that our culture conditions maintained pre-existing antigen-specific memory T cells, although preferentially expanded T cell clones comprised of >  = 2 cells prior to expansion (Supplementary Methods, Supplementary Fig. 13), we decided to perform a 7-day T cell expansion on PBMC from this donor to enable a robust dextramer versus RAPTER comparison. After pre-expanding this donor’s PBMC with the 21 non-control peptides in Supplementary Table 2, we distributed the sample and performed pooled dCODE dextramer and RAPTER assays in parallel (Fig. 5A,B and Supplementary Fig. 14). We compared the TCR sequences identified in the pre- versus post-expansion dCODE dextramer screens and observed significant overlap in clones that stained for dextramers, particularly for TCR clone sizes > 1 and > 10 (Supplementary Fig. 14C). Of the 23 dextramers tested, T cells specific for three haplotype-matched reactivities (CMV pp65, EBV BMLF1, and Influenza M) represented most of the signal in each assay and provided sufficient TCRs to perform correlative analyses. More unique T cell clones were detected by dextramers than by RAPTER, but when the dextramer^+^ and RAPTER^+^ TCR were correlated by clone size, significant overlap among shared TCR clones >  = 5 cells was observed (Fig. 5C).

### Identification of HPV16-specific T cell reactivity using RAPTER

**Figure 6:**
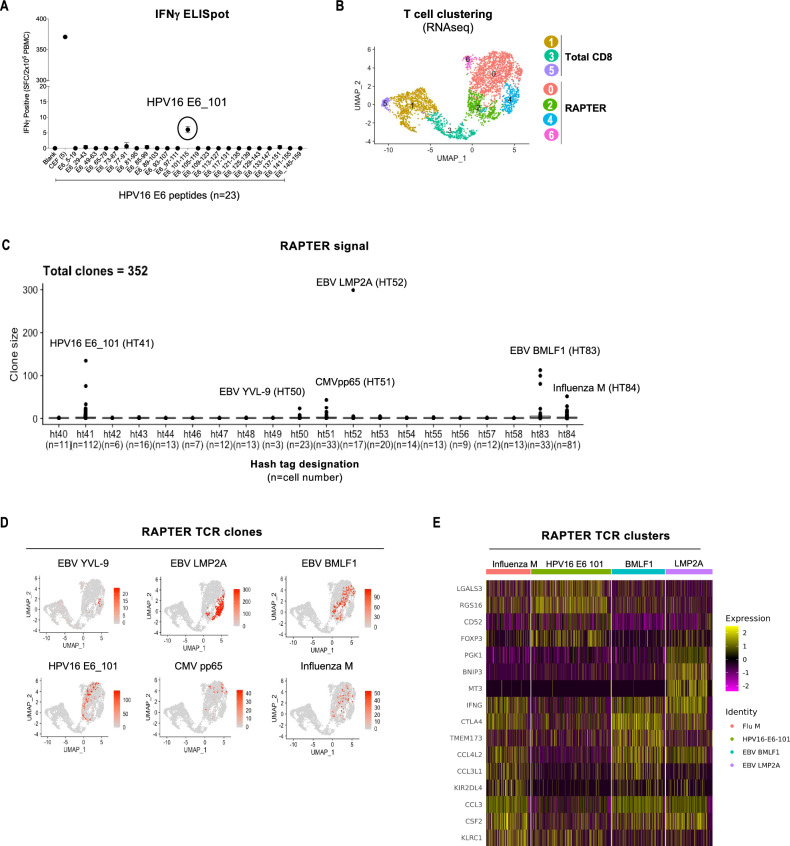
Viral memory T cell detection in a cervical patient blood sample. (**A**) 30 × 10^6^ PBMCs from an HLA-A*02:01^+^ patient with early-stage, treatment-naïve cervical cancer were tested in an IFNγ ELISPOT assay for reactivity against a panel of human papilloma virus (HPV16) E6 15-mer synthetic long peptides (SLP) and 5 HLA-A*02:01-restricted CEF 9-mer peptides. The number of IFNγ spots per 2 × 10^5^ PBMC is shown. (**B**) Unexpanded PBMC (7 × 10^6^ PBMC per assay well) from the patient in (A) were tested in RAPTER to identify HPV16 E6 and CEF peptide specific CD8^+^ T cells. UMAP of total sorted CD8^+^ T cells and CD137/4-1BB^+^ T cells from the RAPTER assay. (**C**) RAPTER signal was demultiplexed by HTO labels to assign individual T cells with cognate peptide reactivities. Out of a total of 3,129T cells, 352 unique TCR clones were identified. TCR clone size (y-axis) is plotted for each HTO designation (x-axis). (**D**) TCR clones identified by RAPTER to be specific for HPV and CEF epitopes are projected onto the scRNA-SEQ UMAP from panel (**B**). The color scale represents the TCR clone size. (**E**) Heat map showing top 16 differentially expressed genes among the Influenza M, HPV16_E6_101, EBV BMLF1, and LMP2A reactive T cell clusters from (**D**).

The immunosuppressive tumor microenvironment can make detecting human papilloma virus (HPV)-specific T cells in the circulation challenging. We wondered if our ability to characterize tumor antigen-specific T cells from patients with HPV16-driven cancer could be improved by RAPTER. To test this, we compared our HPV16 IFNγ ELISpot assay to RAPTER. PBMC were collected from an HLA A*02:01^+^ patient with early-stage cervical cancer at the time of tumor resection but prior to systemic therapy. To determine whether any HPV16 epitope-specific T cells could be detected in this sample, we performed a direct ex vivo IFNγ ELISPOT using three pools of overlapping 15 amino acid-length synthetic long peptides (SLPs) encoding the HPV16 E6, E7, and L1 proteins (Supplementary Table 3). A low-positive HPV16 peptide-specific IFNγ ELISPOT signal was detected (~ 10 SFC/2 × 10^5^ PBMC), which was isolated to the E6 peptide pool (Supplementary Fig. 15, Supplementary Table 3). We performed a second IFNγ ELISPOT using 23 arrayed E6 SLPs and identified a single low-reactive signal against the HPV16 E6_101-115_ (KPLCDLLIRCINCQK) peptide (Fig. 6A, Supplementary Table 4). However, this reactivity was too low (~ 5 SFC/2 × 10^5^ PBMC) to qualify as a true positive.

To assess if the RAPTER assay could provide a stronger signal and greater specificity resolution than the HPV IFNγ ELISPOT, we tested the HPV16^+^ PBMC sample using 15 HPV16 E6 and E7 SLPs, and 5 individual 9 amino acid CEF peptides that comprised the positive control peptide pool (Supplementary Table 5). RAPTER revealed distinct, expanded CD8^+^ T cell clones that reacted to the HPV16 E6_101-115_ (KPLCDLLIRCINCQK) SLP, but no other HPV peptide-specific T cells were identified (Fig. 6C). Additionally, we observed that the CEF control peptide reactivities were predominantly comprised of CD8^+^ T cell reactivities against EBV LMP2A, EBV BMLF1, and Influenza M antigens.

We next compared the phenotypic signatures of the total CD8^+^ T cell population and CD137/4-1BB RAPTER sorted subset. We used scRNA-SEQ data to cluster total CD8^+^ T cells and RAPTER^+^ T cells (Fig. 6B) and noted that RAPTER^+^ T cells segregated transcriptionally into clusters that were dominated by cytotoxic (IFNG, GZMA/B) and activation-associated (41BB, HLA-DR, LAG3) gene programs (Supplementary Fig. 16). Surprisingly, the AIM^+^ HPV and EBV TCR clones clustered separately from each other within these transcript-based functional clusters (Fig. 6D, Supplementary Fig. 16). We selected cells with the four most abundant reactivities corresponding to HPV16_E6_101, EBV BMLF1, EBV LMP2A, and Influenza M, and performed differential gene expression (DEG) analysis (Fig. 6E). Consistent with an immunosuppressive tumor microenvironment, the HPV16_E6_101-specific T cells expressed factors associated with immune suppression including FOXP3, KLRC1 (NKG2A), and LGALS3 (galectin 3) and low levels of AIMs including CCL3, CCL4, and IFNG compared to the other virus-specific clusters, suggesting that the HPV16_E6_101-specific T cells might not be able to optimally respond to cognate ligand stimulation. We therefore show that RAPTER can detect T cell reactivities, even without maximum T cell stimulation.

### Functional validation of the EBV and influenza TCRs identified by RAPTER

We wanted to ensure that RAPTER was reliably detecting different antigen-specific populations, particularly for T cells that are phenotypically very similar. To confirm the specificity of reactive TCRs identified by RAPTER, we cloned the EBV BMLF1 and influenza M TCRs identified by RAPTER and performed functional reactivity tests. We chose these two reactivities since their cognate peptide sequences share similar features (BMLF1: GLCTLVAML; influenza M: GILGFVFTL) and their phenotypic profiles were similar (Fig. 6E). Using the RAPTER scTCR-seq paired chain information from Fig. 6B (Supplementary Table 6), TCR sequences were cloned into a Jurkat T cell reporter line (Fig. 4E). Additionally, we cloned two “non-specific” background TCRs that were found in multiple wells at very low frequency in the RAPTER assay.

**Figure 7:**
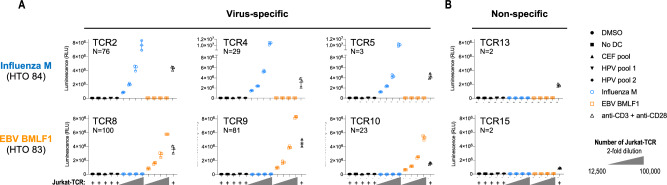
Functional validation of EBV- and influenza A-specific TCRs identified by RAPTER. TCR sequences identified in Fig. 6 that correspond to influenza A epitope (GILGFVFTL HTO84) and EBV BMLF1 epitope (GLCTLVAML, HTO83) were cloned and functionally tested in a Jurkat-TCR luciferase reporter assay. (**A**) Luciferase activity in Jurkat-TCR cells expressing Influenza M (GILGFVFTL) or EBV BMLF1 (GLCTLVAML) epitope-reactive TCRs. The relative abundance of each TCR from the RAPTER assay is indicated and the specific clone frequency is provided under the TCR name. Luciferase activity was measured in Jurkat-TCR cultured alone or Jurkat-TCR co-cultured with dendritic cells pulsed with indicated peptides. (**B**) Luciferase activity in Jurkat-TCR cells engineered with two non-specific background TCRs that were associated with multiple HTO at low frequencies.

A titration of the TCR-engineered Jurkat reporter cells were co-cultured with moDC treated with DMSO, a pool of CEF peptides, a pool of HPV peptides, and the BMLF1 or influenza M test peptides (Supplementary Table 7). All three influenza M TCRs were specifically reactive only to the influenza M peptide in the TCR validation experiment (Fig. 7A), including the low-abundance TCR6 detected in just three cells in the RAPTER assay. Likewise, all three BMLF1 TCRs were reactive specifically to the BMLF1 peptide. Both non-specific TCRs were unreactive to all peptide stimulations tested (Fig. 7B). These experiments provide orthogonal support that the peptide-specific T cell reactivities determined using the RAPTER are accurate.

## Discussion

Here, we describe a method that employs hashing reagents and T cell activation markers to isolate specific T cell-epitope pairs using scRNA-SEQ. We developed RAPTER to simultaneously map the topography of antigen reactivities and identify TCR-epitope couples while using realistic volumes of patient blood. Multiplexing with generic hash tags does not alter standard functional assay culture methods, but does enable pooled sorting and downstream bioinformatic demultiplexing, which significantly improves the detection of low-frequency T cell reactivities. It is essential to choose a hashing target that is not regulated in response to T cell activation, and anti-CD2 proved to be a useful target for the experiments reported here. Alternatively, target-agnostic lipid-based hashing reagents can be used. In this work, we focused on developing RAPTER to identify CD8^+^ T cell TCR-epitope pairs and determined that CD137/4-1BB expression was the most specific AIM that identifies antigen-specific T cells in our culture conditions. We anticipate other AIMs to serve as surrogate markers for antigen specific CD4^+^ T cells, such as CD200 and OX40.

RAPTER successfully deconvoluted specific TCR-epitope reactivities at frequencies as low as 0.15% of total CD8^+^ T cells (0.07% total CD3^+^ cells). This high sensitivity potentially enables the detection and characterization of solid tumor antigen-specific T cell populations that are present at low frequencies in the circulation^24^. To further expand the number of antigen-specific T cells from limited PBMC that RAPTER could capture, we demonstrated that rapid T cell expansion prior to RAPTER maintained existing clonal memory T cell clones and maintained the relative ratios of these reactivities. Our culture conditions preferentially maintained and expanded T cells that were present at clone sizes >  = 2 cells prior to expansion, so if antigen-specific pre-expansion is required to study a sample then the TCR repertoire will likely be skewed and transcriptome data are less relevant due to the effects of peptide pre-expansion on gene expression.

To confirm that TCR-epitope pairs recovered by RAPTER were antigen specific, we cloned several TCRs that were identified by RAPTER to be specific for MART1, EBV BMLF1 and Influenza M epitopes into an engineered Jurkat reporter cell line. Since the standard RAPTER output provides complete, TCRalpha/beta information, we directly cloned TCR sequences of interest for functional validation, highlighting the potential value of rapidly moving from RAPTER screening to TCR validation. RAPTER TCRs were specific for their intended antigen target and not any of the other antigens provided in the RAPTER test, and “non-specific” background TCR sequences had no specific activity. Considering all our data, we suggest selecting RAPTER TCR clone sizes >  = 5 cells for functional validation.

Total input PBMC for RAPTER will depend on the number of reactivities to be tested. In the sensitivity test in Fig. 4, 50,000 total PBMC were seeded into each dilution test well. If we consider the 5^th^ dilution point in which we expected 0.15% of CD8^+^ T cells to be MART1-specific and back-calculate the fraction of CD3^+^ T cells (~ 65% of total CD45^+^) and CD8^+^ T cells (~ 80% of total CD3^+^), we approximated that ~ 39 total MART1^+^ cells would have theoretically been present in this assay well. The RAPTER experiment recovered 22 MART1^+^ TCRs. Therefore, if the number of CD8^+^ T cells in a sample is known, then the number of cells needed to detect reactivities at frequencies >  = 0.15% can be approximated and the assay scaled accordingly.

In general, we observed strong agreement between antigen-specific T cell clones identified independently by RAPTER and dextramer staining and noted that each T cell enrichment method identified TCRs that were not identified by the other. We expected to capture dextramer^+^ TCRs not present in RAPTER since dextramer reagents only measure TCR-epitope binding while RAPTER selects T cells that functionally respond to ligand engagement but were surprised to observe RAPTER TCRs that did not bind dextramer. This might be due to a lack of intact interactions between CD8 and the alpha 3 domain of HLA-I (a potential limitation of the dCODE dextramer technology), HLA supertype reactivities in which a T cell recognizes the same peptide in the context of multiple HLA alleles, or other factors^25,26^.

One key advantage of RAPTER is that it can provide transcriptome, complete TCR sequence, cell surface protein expression, and antigen specificity for each T cell. For example, we found that HPV16_E6_101-specific T cells provided significantly more proportional signal in RAPTER than in IFNγ ELISpot and expressed lower levels of IFNγ transcript and more inhibitory marker transcripts than other reactivities. In this patient sample, HPV-specific T cells might have reduced cytotoxic functionality due to cervical disease, potentially explaining the low ELISpot signal. We were also struck by the distinct phenotypic profiles of the TCR clusters associated with other viral memory reactivities in these patients. All this information was achieved by testing only 7 × 10^6^ unexpanded PBMC in RAPTER.

We envision RAPTER to be compatible with primary cells or cell lines from any species for which HTO (antibody- or lipid-based) and sorting reagents are available. In principle, any antigen format could be used as the source of antigen-specific T cell activation. In this work, we focus on resolving CD8^+^ T cell reactivities, but expect RAPTER could identify CD4^+^ T cell reactivities if the appropriate CD4^+^ T cell AIMs were used. A potentially more scalable version of RAPTER could use an APC cell line that is engineered to express many distinct antigens in an arrayed format. A higher-plex version of RAPTER would require more HTO reagents, but combinatorial hashing might drastically increase RAPTER scalability. RAPTER is also compatible with any oligo-tagged reagent, and we have performed RAPTER assays that incorporate HTO, CITE-seq antibodies, and dCODE dextramers in the same pooled sample (data not shown).

In conclusion, the paired antigen reactivity and TCR information provided by RAPTER can be used to understand the breadth of antigen-specific T cell reactivities in human samples and has the potential to rapidly inform vaccine antigen inclusion and develop targeted TCR therapies.

## Methods

### Use of human samples statement

All methods using human samples were performed in accordance with institutional guidelines and regulations. All human samples were collected after patients and/or their legal guardians provided informed consent. Blood samples collected from human subjects at the New York Medical College were obtained and processed in accordance with a protocol reviewed and approved by the Institutional Review Board at the New York Medical College (IRB Human Subjects Electronic Research Application NYMC_12670). Blood samples purchased from Precision for Medicine were obtained and processed in accordance with their institutional guidelines.

#### Human Peripheral Blood Mononuclear Cells (PBMCs)

Cryopreserved PBMCs were purchased (Precision for Medicine Frederick, Maryland) or isolated from fresh blood from human subjects collected at the New York Medical College. PBMCs were isolated by density gradient centrifugation using Ficoll-Paque Plus (GE Healthcare Life Sciences, #45–001-749) reagent as per manufacturer’s instructions and cryopreserved in freezing media (90% Human Serum AB (Millipore Sigma, #H3667), 10% DMSO (BioPerformance Certified, Millipore Sigma, #D2438).

#### Peptides

Peptides were custom synthesized at Genscript (Piscataway, NJ). Lyophilized peptides were reconstituted in DMSO at 10–50 mg/mL for stock solutions and then further diluted to 10 µg/mL in appropriate assay medium for use. The CEF Control Peptide Pool (Anaspec, #AS-61036–003) was used at 10 µg/mL. Cell Stimulation Cocktail (ThermoFisher, #00–4970-93) was used as per manufacturer’s instructions.

#### Primary Cell Cultures

Cryopreserved PBMCs were thawed and incubated in CellGenix GMP DC serum-free media (CellGenix, #20,801–0500) with 5% Human Serum AB (Millipore Sigma, #H3667) and 1% penicillin–streptomycin- L-glutamine (ThermoFisher Scientific, #10,378–016). Cultures were supplements with dendritic cell and T cell supportive cytokines IL-7 and IL-15 at 5 ng/ml (CellGenix, #1410–050 and #1413–050, respectively), and IL-2 at 10 U/ml (Peprotech, #200–02).

#### Generation of oligo-tagged hashing antibodies

Monoclonal anti-human CD2 antibodies (Clone RPA-2, Biolegend) were conjugated to hashing oligoes (IDT) by inverse electron demand Diels–Alder (iEDDA) click chemistry as previously described^15,27^. Hashtag oligos were ordered with 5’-amine modification (\5AmMC12\ GTGACTGGAGTTCAGACGTGTGCTCTTCCGATCTXXXXXXXXXXXXXXXCCCATATAAGA*A*A, X denotes 15nt barcode unique to each hashtag), and purified by ethanol precipitation upon receipt. Trans-cyclooctene-PEG4-NHS (TCO; Click Chemistry Tools, USA) was dissolved in dry DMSO to 100 mM and added to purified oligos at a tenfold excess in 1X borate buffered saline (BBS, 50 mM borate, 150 mM NaCl, pH 8.5) supplemented with DMSO such that the final DMSO concentration in the reaction mixture is 20%. After 15 min at room temperature, a second aliquot of TCO at tenfold excess was added, and the reaction mixture incubated for another 15 min at room temperature. Residual NHS groups were quenched by the addition of glycine, and the modified oligo was purified by desalting using Micro Bio-Spin P6 columns (Bio-Rad, USA). Antibodies were buffer exchanged into 1X BBS using an Amicon Ultra-4 30 kDa MWCO centrifugal filter (Millipore, USA). Methyltetrazine-PEG4-NHS ester (Click Chemistry Tools, USA) was dissolved in dry DMSO and added at a 30-fold excess to the antibody. The reaction was left for 30 min at room temperature. Residual NHS groups were quenched by the addition of glycine and residual free label was removed via centrifugal filtration. Antibody-oligo conjugates were formed by mixing the modified antibody and oligo at a ratio of 30 pmol of oligo per ug of antibody and incubating at room temperature for at least 1 h, followed by overnight incubation at 4 degrees. Residual methyltetrazine groups on the antibody were quenched by the addition of trans-cyclooctene-PEG4-glycine to 1 mM. Unreacted oligo was removed by centrifugal filtration using an Amicon Ultra-4 50 kDa MWCO filter (Millipore, USA). Purified antibody-oligo conjugate was run on a 4% agarose E-gel (ThermoFisher, USA) to verify conjugation and removal of free oligo. The antibody-oligo conjugate was stored at 4 degrees in PBS (pH 7.2) containing 0.09% sodium azide and 1 mM EDTA.

#### Direct ex vivo IFNγ/Granzyme B ELISPOT

Dual Human IFNγ/GranzymeB FluoroSpot assays kits were purchased from ImmunoSpot (Cleveland, OH) and used per manufacturer’s protocol. Briefly, PBMCs were thawed and incubated in 200 µL in the FluoroSpot plates at 200,000 cells per well with peptide stimulation for 48 h. ELISPOT reactivity was read out on an ImmunoSpot Analyzer using manufacturer’s automated software.

#### Antibodies and phenotypic characterization of T cells by flow cytometry

Fluorescently labeled antibodies were purchased from commercial vendors. To perform flow cytometry phenotypic characterization, cells were harvested, washed, and stained with LIVE/DEAD Green fixable viability dye (Invitrogen cat#L34970). For staining of surface antigens, cells were resuspended in flow cytometry staining buffer (Stain buffer BSA, BD Biosciences, cat#554,657) containing Human Seroblock Fc blocking reagent (BioRad, #BUF070B), incubated for 10 min at room temperature, then fluorescently labeled antibodies of interest were added. Cells were incubated for 30 min at 4 °C and then washed three times with staining buffer. For detection of intracellular proteins, cells were fixed and permeabilized using eBioscience FoxP3/Transcription Factor Staining Buffer Set (Invitrogen #00–5523-00) as per manufactures instructions. Cells were stained for intracellular proteins using fluorescently labeled antibodies diluted in permeabilization buffer for 30 min at 4 °C in dark. Cells were washed prior to acquisition on an A3 Symphony flow cytometer (BD Biosciences). Flow cytometry data were analyzed using the FlowJo v10 (version 10) analysis software (FlowJo, Ashland, OR; https://www.flowjo.com/solutions/flowjo). The following antibodies were purchased from BD Biosciences: Granzyme B Alexa Fluor 647 (clone GB11, #560,212), CD27 APC-R700 (clone M-T271, #565,116), CCR7 BB700 (clone 3D12, #566,437), CD95 BUV395 (clone DX2, #740,306), CD8 BUV496 (clone RPA-T8, #612,942), CD4 BUV563 (clone SK3, #612,912), CD3 BUV661 (clone SK7, #741,692), Tim-3 BUV737 (clone 7D3, #748,820), CD45RA BUV805 (clone HI100, #742,020), GITR BV711 (clone V27-580, #747,662), CD69 BV750 (clone FN50, #747,522), HLA-DR/DP BV786 (clone G46-6, #564,041), CX3CR1 PE-CF594 (clone 2A9-1, #565,897), CD28 PE-Cy5 (clone 8,124,737, #555,730), TCF-7 BB630 (clone S33-966, BD Customs, #624,294), CTLA-4 BB660 (clone BNI3, BD Customs, #624,295), Ki67 BB790 (clone B56, BD Customs, #624,296), HLA-ABC BV570 (clone G46-2.6, BD Customs, #624,298). The following antibodies were purchased from BioLegend: LAG-3 APC/Fire 750 (clone 7H2C65, #369,214), TIGIT BV421 (clone A15153G, #372,710), ICOS BV510 (clone C398.4.A, #313,525), CD45RO BV605 (clone UCHL1, #304,238), 4-1BB BV650 (clone 4B4-1, #309,828), PD-1 PE (clone EH12.2H7, #329,906). The following antibodies were purchased from Invitrogen: CD25 PE-Cy5.5 (clone PC61.5, #35–0251-82), FoxP3 PE-Cy7 (clone PCH101, #25–4776-42).

#### Expansion of antigen-specific T cells

PBMCs were seeded to culture plates in T cell media supplemented with dendritic cell supporting factors GM-CSF at 1000U/mL (CellGenix, #1412–050) and IL-4 at 500 U/mL (CellGenix, #1403–050), T cell supporting cytokines IL-7 at 5 ng/mL (CellGenix, # 1410–050), IL-15 at 5 ng/ml (CellGenix #1413–050), and IL-2 at 10 U/ml (Peprotech, #200–02). Individual peptides or pools of peptides were added to cells at 10 µg/ml (Genscript). Medium containing cytokines was replenished every 2 to 3 days. Cells were re-stimulated with the individual peptides or peptide pools they were initially stimulated with or re-stimulated with DMSO, a control CEF peptide pool or anti-CD3 (clone HIT3a)/anti-CD28 (clone CD28.2) and analyzed 24 h later by flow cytometry for activation induced markers. The antibodies used for flow cytometry analysis were described in Antigen-specific T cell reactivity assays.

#### Antigen-specific T cell reactivity assays

PBMC were seeded to culture plates in T cell media supplemented with dendritic cell supporting factors GM-CSF at 1000U/mL (CellGenix, #1412–050) and IL-4 at 500 U/mL (CellGenix, #1403–050), T cell supporting cytokines IL-7 at 5 ng/mL (CellGenix, #1410–050), IL-15 at 5 ng/ml (CellGenix #1413–050), and IL-2 at 10 U/ml (Peprotech, cat# 200–02). Individual peptides of interest were added to unique assay wells at 10 µg/ml (Genscript). T cell reactivity was analyzed either 24 h post-peptide stimulation or after 10-day expansion. For 10-day expansion cultures, cells were fed with fresh media and cytokines every two days for 10 days after the initial peptide addition. Then, individual peptides of interest were added to T cell expansion cultures for overnight re-stimulation to upregulate 4-1BB expression and enable antigen-specific T cell sorting. Following peptide re-stimulation, cells were prepared for either flow cytometry characterization or were further processed to enable hashing, pooling, and single cell sequencing. Antibodies used for flow cytometry analysis were purchased from commercial vendors. The following antibodies were purchased from BD Biosciences: CD3 BUV661 (clone SK7, #741,692), CD8 BUV496 (clone RPA-T8, #612,942), CD4 BUV563 (clone SK3, #612,912), CD69 BV750 (clone FN50, #747,522), HLADR BUV395 (clone DX2, #740,306), Ki67 BB790 (clone B56, BD Customs #624,296). The following antibodies were purchased from BioLegend: 4-1BB BV650 (clone 4B4-1, #309,828), PD-1 PE (clone EH12.2H7, #329,906), CD25 Alexa Fluor 488 (clone M-A251, #356,116), IFNγ PE-Cy7 (clone 4S.B3, #502,528), CX3CR1 PE/Dazzle594 (clone 2A9-1, #341,623), CD40L BV421 (clone 24–31, #310,824), OX40 APC (clone Ber-ACT35, #350,008).

#### Cell hashing following functional T cell assay performance

Following functional stimulation, cells from individual assay wells were collected into a 96 well assay block, washed, and resuspended in flow cytometry BD BSA staining buffer (BD Biosciences, #554,657) containing hashing reagents of interest. Cells were either stained with one or two hashing antibodies, each at 1 µg/1 million cells. Cells were incubated for 30 min at 4 °C, washed twice, then pooled. If oligonucleotide-tagged dextramers were included in the analysis, then samples were stained with dextramers before proceeding to CITE-seq and flow cytometry antibody staining as per the oligo-tagged dextramer staining protocol below.

#### CITE-seq antibody staining and fluorescent antibody staining

Following the hashing staining procedure, pooled and hashed samples were resuspended in BD BSA staining buffer containing both CITE-seq antibodies as well as fluorescently tagged flow cytometry antibodies at their respective optimal concentrations. Cells were incubated for 30 min at 4 °C, washed twice, and then sorted for single cell sequencing.

#### Oligo-tagged dextramer staining and FACS sorting

Cryopreserved health donor PBMC were thawed briefly in a 37 °C water bath. CD8^+^ T cells were enriched using magnetic beads (Miltenyi Biotec). Cells were washed by centrifugation and then treated with PBS (Gibco, #14190-250) containing benzonase (Millipore, #70664) and 50 nM Dasatinib (Axon Medchem, #1392) for 45 min at 37 °C. Cells were transferred to a 96-well assay block (Corning, #3960), centrifuged, and supernatant was aspirated. The appropriate custom Immudex dCODE-PE dextramer pool (Copenhagen, Denmark) was added at 1 µl/100 µl reaction for 30 min in dark at room temperature. Next, the fluorochrome-labeled surface markers were added, and the cells were incubated for additional 30 min at 4 °C. After washes, the cells were immediately sorted. Flow cytometry antibody staining, and washes were performed in staining buffer (BD, #554657). Surface markers for FACS included the following markers and fluorophores: Live/Dead—DAPI (Sigma, #10236276001), CD3 BUV737 (BD Biosciences, #612750), CD4 BV510 (BD Biosciences, #563919), CD8 BUV805 (BD Biosciences, #612889), CCR7 AF647 (BioLegend #353218), and CD45RO BV605 (BioLegend #304238).

#### 4-1BB + T cell FACS sorting

Twenty-four hours following re-stimulation, cells were collected and stained with fluorescently-labeled antibodies for FACS using an Astrios cell sorter (Beckman Coulter) using the following surface antibodies: CD3 (BD Biosciences, #612750), CD8 (BD Biosciences, #612889), CD69 (BD Biosciences, #564364), CCR7 (Biolegend, #353218), CD45RO (Biolegend, #304238), CD137 (Biolegend, #309828), and CD25 (Biolegend, #356104). Gates for forward scatter plot, side scatter plot, and fluorescent channels were set to select live cells while excluding debris and doublets. A 100 µm nozzle was used to sort single CD3 + CD8 + CD45RO + CD137 + cells for further processing.

#### Chromium single cell partitioning and library preparation

Sorted cells were loaded onto a Chromium Single Cell 5’ Chip (10× Genomics, 1000287) and processed through the Chromium Controller to generate GEMs (Gel Beads in Emulsion). RNA-Seq libraries were prepared with the Chromium Single Cell 5’ Library & Gel Bead Kit (10× Genomics, 1000265) following the manufacturer’s protocol.

#### Plasmid cloning

TRA and TRB of EBV BMFL1 or Flu M TCRs were subcloned into the lentiviral vector pLVX-EF1a at Genscript. TRA and TRB of MART1 TCRs were subcloned into the lentiviral vector pLVX-EF1a IRES-EGFP between SpeI and XbaI.

#### Transfection and lentiviral production

Lenti-X 293T cells were seeded on tissue culture plates coated with poly-D-lysine. Transfection was done using Lenti-X Packaging Single Shots (Takara) as per manufacturer’s instruction. Supernatant containing lentiviral particles was collected 48 h and 72 h post transfection. When needed, viral supernatant was concentrated using Amicon Ultra-15 centrifugal Filter Unit (NMWL 100 kDa. Millipore UFC910096).

Jurkat reporter cell lines: Jurkat/NFAT-Luc cell lines were generated by stable transfection of Jurkat (Clone E6-1, ATCC) with an NFAT response element-luciferase reporter plasmid. Jurkat TCRab dKO cell line was generated by CRISPR editing of the TRA and TRB in the Jurkat Clone E6-1 (ATCC TIB-152). TRB was edited using a single gRNA (AGGCTTCTTCCCCGACCACG). TRA was edited using two gRNAs (ACATACCAGAAGAGATATGG and TGGATTTAGAGTCTCTCAGC. Lentiviral particles expressing an AP-1 response element-firefly luciferase (Qiagen) were used to transduce Jurkat TCRab dKO cells to generate Jurkat TCRab dKO/AP1.Luc cells, which were subsequently transduced with lentiviral particles expressing human CD8 in pLVX. The resulting Jurkat TCRab dKO/AP1.Luc/hCD8 cells were used to assess antigen reactivity of TCRs. Briefly, lentiviral particles harboring the TRA and TRB of a TCR of interest were transduced into Jurkat TCRab dKO/AP1.Luc/hCD8 in the presence of 8 μg/mL polybrene. TCR expression was confirmed by flow cytometry analyses using the following antibodies: CD3 BUV661 (Clone SK7, BD Biosciences), CD4 BUV563 (Clone SK3, BD Biosciences), CD8 BUV496 (Clone RPA-T8, BD Biosciences) and TCRa/b PerCP-vio700 (Clone REA652, Miltenyi Biotec). When needed, TCR^+^ cells were enriched by anti-APC microbeabs (Miltenyi Biotec) after labeling cells with an APC-conjugated anti-human TCRab antibody (Clone REA652, Miltenyi Biotec).

#### Jurkat firefly luciferase reporter assays

CD14^+^ cells isolated from PBMC using EasySep Human Monocyte Isolation Kit (STEMCELL) were cultured in CellGenix GMP DC serum-free media (CellGenix, #20801-0500) supplemented with 1% Human Serum AB (Millipore Sigma, #H3667) and 1% penicillin–streptomycin-L-glutamine (ThermoFisher Scientific, #10378-016). GM-CSF (1000 U/mL, CellGenix #1412-050) and IL-4 (500 U/mL, CellGenix #1403-050) were added to the medium to generate Mo-DC. On day 5, DC were pulsed with peptide pools or individual peptides at 10 μg/mL, in the presence of IFNα (500U/mL), GM-CSF (1000 U/mL) and IL-4 (500 U/mL). 12,500 to 100,000 Jurkat TCRab dKO/AP1.Luc/hCD8 cells expressing an TCR of interest (Jurkat-TCR) were cocultured with 30,000 DC pulsed with antigen peptides for 5 h before lysed to assess luciferase activity using One-Glo Luciferase Assay (Promega) as per manufacturer’s instructions. Co-culture of 100,000 Jurkat-TCR cells with 30,000 DC pulsed with irrelevant peptides or with DC treated with DMSO was used as negative controls. When a titration of Jurkat-TCR cells were used, Jurkat/NFAT-Luc cells were used as filler cells. Nonspecific activation of TCRs was achieved by soluble anti-CD3 (Clone HIT3a, BD Biosciences) and anti-CD28 (Clone CD28.2, BioLegend). Luminescence signal was measured using the EnVision plate reader. RLU values were plotted after background signals were subtracted.

### Bioinformatic methods

The transcriptome, TCR (VDJ), hashing, CITE-seq, and dextramer libraries were sequenced and the raw sequencing data was processed using the 10X CellRanger analysis pipeline. Mapped reads were prepared for public distribution by removing patient genetic information with bamboozle software^28^. The CellRanger analysis generated feature-barcode UMI count matrices and TCR(VDJ) amino acid sequences. The features include gene expression, hashing antibody, CITE-seq antibody, and dextramer capture. Using the feature-barcode matrices as the input, the R package Seurat v3.1.4^29^ was used for downstream analysis. Standard log normalization of gene UMI counts was performed, followed by identification of 1000 most variable genes, and scaling and centering of the data. Next Principal Component Analysis (PCA) was performed, and 50 PCs were computed and stored. Clustering was then performed using Seurat’s graph-based clustering approach. A k-nearest neighbor (KNN) graph was computed based on the Euclidean distance in a 20-dimensional PCA space followed by clustering at various resolutions. At each resolution, top marker genes were identified and used to create a heatmap of gene expression across different clusters. Upon visual inspection, the optimal clustering resolution was determined. All the cells belonging to the dead cell cluster, with mitochondrial genes as the top gene markers, were removed from the downstream analysis. We also removed cells for which number of genes detected was less than or equal to 500, and fraction of mitochondrial gene expression was greater than or equal to 0.25. Since one of the main goals of the assay is to identify T-cell reactivity against various antigens which are driven by TCR-antigen interactions, we removed any cell with a single TCR chain, or a non-productive chain, or more than 1 alpha or beta chain. Any outlier cell with large number of genes detected and/or many UMIs detected was also removed. For the remaining cells, data from other features (CITE-seq, hashing, dextramer) was then processed. The data from count matrices corresponding to those features was normalized using centered log ratio transformation, and then scaled. Hashing data was used to demultiplex the cells using the MultiSeqDemux algorithm default parameters^30^. Any cell that was not assigned a hashtag according to the hashing scheme was removed after multiplexing. For each cell, we have the paired TCR amino acid sequence which defines the unique functional clonotype of the cell. After demultiplexing, we calculated the clonotype size of each T-cell clone among all the cells associated with a hash tagged assay well. Any clonotype with size > 20 was considered to have potential reactivity to the specific antigen in the hash tagged well.

## Supplementary Information

Supplementary Information 1.

Supplementary Information 2.

## Data Availability

All sequencing data are available on GEO under the accession number GSE231977 (Supplementary Table 8).
