# Supplementary material for: Rapid TCR:Epitope Ranker (RAPTER): a primary human T cell reactivity screening assay pairing epitope and TCR at single cell resolution
Source: Sci Rep. 2023 May 25;13:8452. doi: 10.1038/s41598-023-35710-7 (PMC10212918; doi:10.1038/s41598-023-35710-7)
Supplement: Supplementary file 1 — Supplementary Information 1. [file 41598_2023_35710_MOESM1_ESM.pdf]

Supplemental Table 1. MART1 TCR sequences from TCR validation in Figure 4F

Location in scSEQ experiment	MART1 TCR ID in validation	Median 41BB expression in RAPTER	TCR sequence (J chains only)
RAPTER and Dextramer	MART1 DR3	1.983	TRAV12-2*01:None:TRAJ6*01:CAVPGGGSYIPTF:TRBV27*01:TRBD2*01:TRBJ2-7*01:CASSPTSGGEPFSYEQYF
	MART1 DR4	1.6384	TRAV12-2*01:None:TRAJ49*01:CAVGLVHTGNQFYF:TRBV6-5*01:TRBD1*01:TRBJ1-2*01:CASSYGTLTADGYTF
	MART1 DR5	2.009939525	TRAV12-2*01:None:TRAJ48*01:CAVALRGNEKLTF:TRBV27*01:TRBD2*01:TRBJ2-2*01:CASSTGTSVFTGELFF
RAPTER only	MART1 R3	1.876606083	TRAV12-3*01:None:TRAJ23*01:CASLSGGKLIF:TRBV3-1*01:TRBD2*02:TRBJ2-1*01:CASSPLAGTGNEQFF
Dextramer only	MART1 D1	N/A	TRAV12-2*01:None:TRAJ40*01:CAVRVEGVF:TRBV6-1*01:TRBD1*01:TRBJ2-3*01:CASKKGQGVFSDTQYF
	MART1 D3	N/A	TRAV12-2*01:None:TRAJ35*01:CAVNLGFGNVLHC:TRBV27*01:TRBD1*01:TRBJ2-3*01:CASSREGGRADTQYF
	MART1 D5	N/A	TRAV12-2*01:None:TRAJ47*01:CAVYDKLVF:TRBV19*01:TRBD1*01:TRBJ1-3*01:CASSLGRLSGNTIYF
Background	MART1 B6	0.975986598	TRAV12-2*01:None:TRAJ49*01:CAVNTGNQFYF:TRBV4-3*01:TRBD2*02:TRBJ2-1*01:CASSQVLLAGAGEQFF

Supplemental Table 2. Dextramers used in Supplementary Figure 11 & Supplementary Figure 12

Source	Gene	Peptide	HLA restriction	HTO barcode
CMV	IE-1	KLGGALQAK	HLA-A*03:01	hTag_53_regn
	pp65	NLVPMVATV	HLA-A*02:01	hTag_48_regn
	IE-1	VTEHDTLLY	HLA-A*01:01	hTag_83_regn
	pp65	RPHERNGFTVL	HLA-B*07:02	hTag_85_regn
EBV	EBNA 3B	AVFDRKSDAK	HLA-A*11:01	hTag_54_regn
	EBNA 3B	IVTDFSVIK	HLA-A*11:01	hTag_56_regn
	EBNA 3A	RLRAEAQVK	HLA-A*03:01	hTag_55_regn
	BZLF1	RAKFKQLL	HLA-B*08:01	hTag_58_regn
	BMLF1	GLCTLVAML	HLA-A*02:01	hTag_47_regn
	LMP2A	FLYALALLL	HLA-A*02:01	hTag_42_regn
	EBNA 3B	LLDFVRFMGV	HLA-A*02:01	hTag_46_regn
	LMP1	YLLEMLWRL	HLA-A*02:01	hTag_49_regn
	EBNA 6	QPRAPIRPI	HLA-B*07:02	hTag_50_regn
Influenza A	Flu MP	GILGFVFTL	HLA-A*02:01	hTag_40_regn
Tumor	MART-1	ELAGIGILTV	HLA-A*02:01	hTag_57_regn
	MAGE-A1	KVLEYVIKV	HLA-A*02:01	hTag_41_regn
	MAGE-A3	KVAELVHFL	HLA-A*02:01	hTag_84_regn
	HPV16 E7	MLDLQPETT	HLA-A*02:01	hTag_44_regn
	NY-ESO-1	SLLMWITQV	HLA-A*02:01	hTag_51_regn
HIV	Gag Protein	SLFNTVATLY	HLA-A*02:01	hTag_43_regn
	Gag Protein	SLYNTVATLY	HLA-A*02:01	hTag_52_regn
Neg Controls	Neg. Ctrl.	ALIAPVHAV	HLA-A*02:01	n/a
	Neg. Ctrl.	AAKGRGAAL	HLA-B*08:01	n/a

Supplemental Table 3. Peptides used in ELISpot for Supplementary Figure 15

Pool name		Source	Peptide name	Peptide Sequence
HPV16 E6, E7, L1 pool (n=32)	HPV16 E6 pool (n=23)	HPV16 E6	HPV16 E6 5	RTAMFQDPQERPRKL
		HPV16 E6	HPV16 E6 29	TIHDIILECVYCKQQ
		HPV16 E6	HPV16 E6 49	VYDFAFRDLCIVYRD
		HPV16 E6	HPV16 E6 65	NPYAVCDKCLKFYSK
		HPV16 E6	HPV16 E6 73_	CLKFYSKISEYRHYC
		HPV16 E6	HPV16 E6 77	YSKISEYRHYCYSLY
		HPV16 E6	HPV16 E6 81	SEYRHYCYSLYGTTL
		HPV16 E6	HPV16 E6 85	HYCYSLYGTTLEQQY
		HPV16 E6	HPV16 E6 89	SLYGTTLEQQYNKPL
		HPV16 E6	HPV16 E6 93	TTLEQQYNKPLCDLL
		HPV16 E6	HPV16 E6 97	QQYNKPLCDLLIRCI
		HPV16 E6	HPV16 E6 101	KPLCDLLIRCINCQK
		HPV16 E6	HPV16 E6 105	DLLIRCINCQKPLCP
		HPV16 E6	HPV16 E6 109	RCINCQKPLCPEEKQ
		HPV16 E6	HPV16 E6 113	CQKPLCPEEKQRHLD
		HPV16 E6	HPV16 E6 117	LCPEEKQRHLDKKQR
		HPV16 E6	HPV16 E6 121	EKQRHLDKKQRFHNI
		HPV16 E6	HPV16 E6 125	HLDKKQRFHNIRGRW
		HPV16 E6	HPV16 E6 129	KQRFHNIRGRWTGRC
		HPV16 E6	HPV16 E6 133	HNIRGRWTGRCMSCC
		HPV16 E6	HPV16 E6 137	GRWTGRCMSCCRSSR
		HPV16 E6	HPV16 E6 141	GRCMSCCRSSRTRRE
		HPV16 E6	HPV16 E6 145	MSCCRSSRTRRETQL
	HPV16 L1 pool (n=4)	HPV16 L1	HPV16 L1 385	ADVMTYIHSMNSTIL
		HPV16 L1	HPV16 L1 389	TYIHSMNSTILEDWN
		HPV16 L1	HPV16 L1 437	KEDPLKKYTFWEVNL
		HPV16 L1	HPV16 L1 441	LKKYTFWEVNLKEKF
		HPV16 E7	HPV16 E7 13	LDLQPETTDLYCYEQ
		HPV16 E7	HPV16 E7 41	PAGQAEPDRAHYNIV
		HPV16 E7	HPV16 E7 57	FCCKCDSTLRLCVQS
		HPV16 E7	HPV16 E7 61	CDSTLRLCVQSTHVD
		HPV16 E7	HPV16 E7 65	LRLCVQSTHVDIRTL
CEF pool (n=5)		CMVpp65	CMVpp65_NLV	NLVPMVATV
		EBV LMP2A	EBV LMP2A_CLG	CLGGLLTMV
		EBV BMLF1	EBV BMLF1_GLC	GLCTLVAML
		EBV YVL9	EBV YVL9_YVL	YVLDHLIVV
		Influenza M	Influenza M_GIL	GILGFVFTL

Supplemental Table 4. HPV peptides used for ELISpot in Figure 6A

Source	Peptide name	Peptide sequence	Length
HPV16 E6	HPV16 E6_5	RTAMFQDPQERPRKL	15
	HPV16 E6_29	TIHDIILECVYCKQQ	15
	HPV16 E6_49	VYDFAFRDLCIVYRD	15
	HPV16 E6_65	NPYAVCDKCLKFYSK	15
	HPV16 E6_73	CLKFYSKISEYRHYC	15
	HPV16 E6_77	YSKISEYRHYCYSLY	15
	HPV16 E6_81	SEYRHYCYSLYGTTL	15
	HPV16 E6_85	HYCYSLYGTTLEQQY	15
	HPV16 E6_89	SLYGTTLEQQYNKPL	15
	HPV16 E6_93	TTLEQQYNKPLCDLL	15
	HPV16 E6_97	QQYNKPLCDLLIRCI	15
	HPV16 E6_101	KPLCDLLIRCINCQK	15
	HPV16 E6_105	DLLIRCINCQKPLCP	15
	HPV16 E6_109	RCINCQKPLCPEEKQ	15
	HPV16 E6_113	CQKPLCPEEKQRHLD	15
	HPV16 E6_117	LCPEEKQRHLDKKQR	15
	HPV16 E6_121	EKQRHLDKKQRFHNI	15
	HPV16 E6_125	HLDKKQRFHNIRGRW	15
	HPV16 E6_129	KQRFHNIRGRWTGRC	15
	HPV16 E6_133	HNIRGRWTGRCMSCC	15
	HPV16 E6_137	GRWTGRCMSCCRSSR	15
	HPV16 E6_141	GRCMSCCRSSRTRRE	15
	HPV16 E6_145	MSCCRSSRTRRETQL	15
CEF	CMVpp65	NLVPMVATV	9
	EBV LMP2A	CLGGLLTMV	9
	EBV BMLF1	GLCTLVAML	9
	EBV YVL9	YVLDHLIVV	9
	Influenza M	GILGFVFTL	9

Supplemental Table 5. Peptides used for RAPTER in Figure 6B

Gene	Peptide name	Peptide sequence	Length	HTO
HPV16 E6	HPV16 E6_29	TIHDIILECVYCKQQ	15	hTag_55_regn
	HPV16 E6_49	VYDFAFRDLCIVYRD	15	hTag_54_regn
	HPV16 E6_65	NPYAVCDKCLKFYSK	15	hTag_47_regn
	HPV16 E6_73	CLKFYSKISEYRHYC	15	hTag_46_regn
	HPV16 E6_77	YSKISEYRHYCYSLY	15	hTag_53_regn
	HPV16 E6_81	SEYRHYCYSLYGTTL	15	hTag_43_regn
	HPV16 E6_85	HYCYSLYGTTLEQQY	15	hTag_44_regn
	HPV16 E6_89	SLYGTTLEQQYNKPL	15	hTag_42_regn
	HPV16 E6_101	KPLCDLLIRCINCQK	15	hTag_41_regn
	HPV16 E6_121	EKQRHLDKKQRFHNI	15	hTag_56_regn
	HPV16 E6_133	HNIRGRWTGRCMSCC	15	hTag_57_regn
HPV16 E7	HPV16 E7_13	LDLQPETTDLYCYEQ	15	hTag_40_regn
	HPV16 E7_57	FCCKCDSTLRLCVQS	15	hTag_58_regn
	HPV16 E7_65	LRLCVQSTHVDIRTL	15	hTag_49_regn
HPV16 L1	HPV16 L1_389	TYIHSMNSTILEDWN	15	hTag_48_regn
CMV	CMVpp65	NLVPMVATV	9	hTag_51_regn
EBV	EBV LMP2A	CLGGLLTMV	9	hTag_52_regn
	EBV BMLF1	GLCTLVAML	9	hTag_83_regn
	EBV YVL9	YVLDHLIVV	9	hTag_50_regn
Influenza	Influenza M	GILGFVFTL	9	hTag_84_regn

Supplemental Table 6. TCR cloned for functional validation in Figure 7

TCR ID	Reactivity to be confirmed	HTO designation	TCR sequence (J chain only)	Cell count in RAPTER
TCR 2	Influenza M	hTag-84-regn construct 2	TRAV27*01:None:TRAJ37*01:CAGGGSGNTGKLIF:TRBV19*01:None:TRBJ2-7*01:CASSVRSSYEQYF	76
TCR 3	Influenza M	hTag-84-regn construct 3	TRAV27*01:None:TRAJ42*01:CAGGGSQGNLIF:TRBV19*01:None:TRBJ2-7*01:CASSIRSAYEQYF	34
TCR 5	Influenza M	hTag-84-regn construct 5	TRAV25*01:None:TRAJ42*01:CAGGWGGSQGNLIF:TRBV19*01:TRBD2*01:TRBJ2-2*01:CASSQRSTGELFF	3
TCR 7	EBV BMLF1	hTag-83-regn construct 1	TRAV12-1*01:None:TRAJ12*01:CVVNGGDSSYKLIF:TRBV2*01:TRBD1*01:TRBJ2-2*01:CASGDGQRAPGELFF	113
TCR 10	EBV BMLF1	hTag-83-regn construct 4	TRAV5*01:None:TRAJ31*01:CAEDNNARLMF:TRBV20-1*02:TRBD1*01:TRBJ1-3*01:CSARVGVGNTIYF	23
TCR 11	EBV BMLF1	hTag-83-regn construct 7	TRAV5*01:None:TRAJ31*01:CAEDNNARLMF:TRBV20-1*02:TRBD1*01:TRBJ2-7*01:CSARAASRGQGAQITYEQYF	3
TCR 13	Non-specific	Non-specific	TRAV12-3*01:None:TRAJ27*01:CAMSDNTNAGKSTF:TRBV20-1*02:TRBD1*01:TRBJ2-7*01:CSARAASRGQGAQITYEQYF	2
TCR 15	Non-specific	Non-specific	TRAV27*01:None:TRAJ52*01:CALAGGTSYGKLTF:TRBV6-5*01:TRBD1*01:TRBJ1-2*01:CASSYWDRIANYGYTF	2

Supplemental Table 7. Peptides used in Figure 7

Control pools	Peptide name	Peptide sequence
HPV pool 1	HPV16 E6 29	TIHDIILECVYCKQQ
	HPV16 E6 49	VYDFAFRDLCIVYRD
	HPV16 E6 65	NPYAVCDKCLKFYSK
	HPV16 E6 73	CLKFYSKISEYRHYC
	HPV16 E6 77	YSKISEYRHYCYSLY
	HPV16 E6 81	SEYRHYCYSLYGTTL
	HPV16 E6 89	SLYGTTLEQQYNKPL
	HPV16 E6 121	EKQRHLDKKQRFHNI
	HPV16 E6 133	HNIRGRWTGRCMSCC
HPV pool 2	HPV16 E6 e18	LLIRCINCQK
	HPV16 E6 26 p1	LCDLLIRCI
	HPV16 E6 26 p2	KPLCDLLIR
CEF pool	EBV YVL-9	YVLDHLIVV
	CMV pp65	NLVPMVATV
	EBV LMP2A	CLGGLLTMV

Supplemental Table 8. GEO accession numbers for single cell RNA sequencing files.

Accession	Title	Data type
**GSE231977**	**Rapid TCR:Epitope Ranker (RAPTER): A primary human T cell reactivity screening assay pairing epitope and TCR at single cell resolution**	**scRNA-SEQ**
GSM7306830	Pre-expanded CD8+ Tc Total	scRNA-SEQ
GSM7306831	Pre-expanded CD8+ CMVpp65+ Multimer Selection	scRNA-SEQ
GSM7306832	Pre-expanded CMVpp65 restim CD8+ 41BB+ Selection	scRNA-SEQ
GSM7306833	Pre-expanded CMVpp65 restim CD8+ PD1+ Selection	scRNA-SEQ
GSM7306834	Pre-expanded CD8+ MART1+ Multimer Selection	scRNA-SEQ
GSM7306835	Pre-expanded MART1 restim CD8+ 41BB+ Selection	scRNA-SEQ
GSM7306836	Pre-expanded MART1 restim CD8+ PD1+ Selection	scRNA-SEQ
GSM7306837	Day 1 sort CD3+, CD8+	scRNA-SEQ
GSM7306838	Day 1 sort CD3+, CD8+ 41BB+	scRNA-SEQ
GSM7306839	Day 8 Pre-expanded sort CD3+, CD8+	scRNA-SEQ
GSM7306840	Day8 Pre-expanded sort CD3+, CD8+ 41BB+	scRNA-SEQ
GSM7306841	Day 7 Pre-expanded sort CD3+ CD8+	scRNA-SEQ
GSM7306842	Day 7 Pre-expanded sort CD3+ CD8+ Dextramer pool+	scRNA-SEQ
GSM7306843	Total Pre-expanded CD3+ T cells	scRNA-SEQ
GSM7306844	Pre-expanded CEF pool, hashed, 4-1BB sorted	scRNA-SEQ
GSM7306845	Pre-Expanded CMVpp65 Cells, CD8+ CMVpp65+ Dextramer sorted (TCR repertoire control), rep1	scRNA-SEQ
GSM7306846	Pre-Expanded CMVpp65 Cells, CD8+ CMVpp65+ Dextramer sorted (TCR repertoire control), rep2	scRNA-SEQ
GSM7306847	Pre-Expanded MART1 Cells, CD8+ MART1+ Dextramer sorted (TCR repertoire control), rep1	scRNA-SEQ
GSM7306848	Pre-Expanded MART1 Cells, CD8+ MART1+ Dextramer sorted (TCR repertoire control), rep2	scRNA-SEQ
GSM7306849	Pre-Expanded CMVpp65 Cells, overnight re-stimulated CD8+ 41BB+ sorted (pooled n = 13, 2 HTOs each)	scRNA-SEQ
GSM7306850	Pre-Expanded MART1 Cells, overnight re-stimulated CD8+ 41BB+ sorted (pooled n = 12, 2 HTOs each)	scRNA-SEQ
GSM7306851	Sorted CD3+CD8+	scRNA-SEQ
GSM7306852	Sorted CD3+CD8+41BB+	scRNA-SEQ
GSM7306853	Full 50 Dextramer Pool Stained, CD3+CD8+ T Cells, TCR Repertoire Control	scRNA-SEQ
GSM7306854	Sorted Full 50 Dextramer Pool Stained, CD3+CD8+ Dextramer+ Pool (n = 50 dextramers), rep1	scRNA-SEQ
GSM7306855	Sorted Full 50 Dextramer Pool Stained, CD3+CD8+ Dextramer+ Pool (n = 50 dextramers), rep2	scRNA-SEQ

GSM7306856	Pre-expanded CD8+ Tc Total, scTCR-seq	scTCR -SEQ
GSM7306857	Pre-expanded CD8+ CMVpp65+ Multimer Selection	scTCR -SEQ
GSM7306858	Pre-expandedCMVpp65 restim CD8+ 41BB+ Selection	scTCR -SEQ
GSM7306859	Pre-expanded CMVpp65 restim CD8+ PD1+ Selection	scTCR -SEQ
GSM7306860	Pre-expanded CD8+ MART1+ Multimer Selection	scTCR -SEQ
GSM7306861	Pre-expanded MART1 restim CD8+ 41BB+ Selection	scTCR -SEQ
GSM7306862	Pre-expanded MART1 restim CD8+ PD1+ Selection	scTCR -SEQ
GSM7306863	Day 1 sort CD3+, CD8+	scTCR -SEQ
GSM7306864	Day 1 sort CD3+, CD8+ 41BB+	scTCR -SEQ
GSM7306865	Day 8 Pre-expanded sort CD3+, CD8+	scTCR -SEQ
GSM7306866	Day8 Pre-expandedsort CD3+, CD8+ 41BB+	scTCR -SEQ
GSM7306867	Day 7 Pre-expanded sort CD3+ CD8+	scTCR -SEQ
GSM7306868	Day 7 Pre-expanded sort CD3+ CD8+ Dextramer pool+	scTCR -SEQ
GSM7306869	Total Pre-expanded CD3+ T cells	scTCR -SEQ
GSM7306870	Pre-expanded CEF pool, hashed, 4-1BB sorted	scTCR -SEQ
GSM7306871	Pre-Expanded CMVpp65 Cells, CD8+ CMVpp65+ Dextramer sorted (TCR repertoire control), rep1	scTCR -SEQ
GSM7306872	Pre-Expanded CMVpp65 Cells, CD8+ CMVpp65+ Dextramer sorted (TCR repertoire control), rep2	scTCR -SEQ
GSM7306873	Pre-Expanded MART1 Cells, CD8+ MART1+ Dextramer sorted (TCR repertoire control), rep1	scTCR -SEQ
GSM7306874	Pre-Expanded MART1 Cells, CD8+ MART1+ Dextramer sorted (TCR repertoire control), rep2	scTCR -SEQ
GSM7306875	Pre-Expanded CMVpp65 Cells, overnight re-stimulated CD8+ 41BB+ sorted (pooled n = 13, 2 HTOs each)	scTCR -SEQ
GSM7306876	Pre-Expanded MART1 Cells, overnight re-stimulated CD8+ 41BB+ sorted (pooled n = 12, 2 HTOs each)	scTCR -SEQ
GSM7306877	Sorted CD3+CD8+	scTCR -SEQ
GSM7306878	Sorted CD3+CD8+41BB+	scTCR -SEQ
GSM7306879	Full 50 Dextramer Pool Stained, CD3+CD8+ T Cells, TCR Repertoire Control	scTCR -SEQ
GSM7306880	Sorted Full 50 Dextramer Pool Stained, CD3+CD8+ Dextramer+ Pool (n = 50 dextramers), rep1	scTCR -SEQ
GSM7306881	Sorted Full 50 Dextramer Pool Stained, CD3+CD8+ Dextramer+ Pool (n = 50 dextramers), rep2	scTCR -SEQ
