# Supplementary material for: Rapid TCR:Epitope Ranker (RAPTER): a primary human T cell reactivity screening assay pairing epitope and TCR at single cell resolution
Source: Sci Rep. 2023 May 25;13:8452. doi: 10.1038/s41598-023-35710-7 (PMC10212918; doi:10.1038/s41598-023-35710-7)
Supplement: Supplementary file 2 — Supplementary Information 2. [file 41598_2023_35710_MOESM2_ESM.pdf]

# Supplementary Figure 1

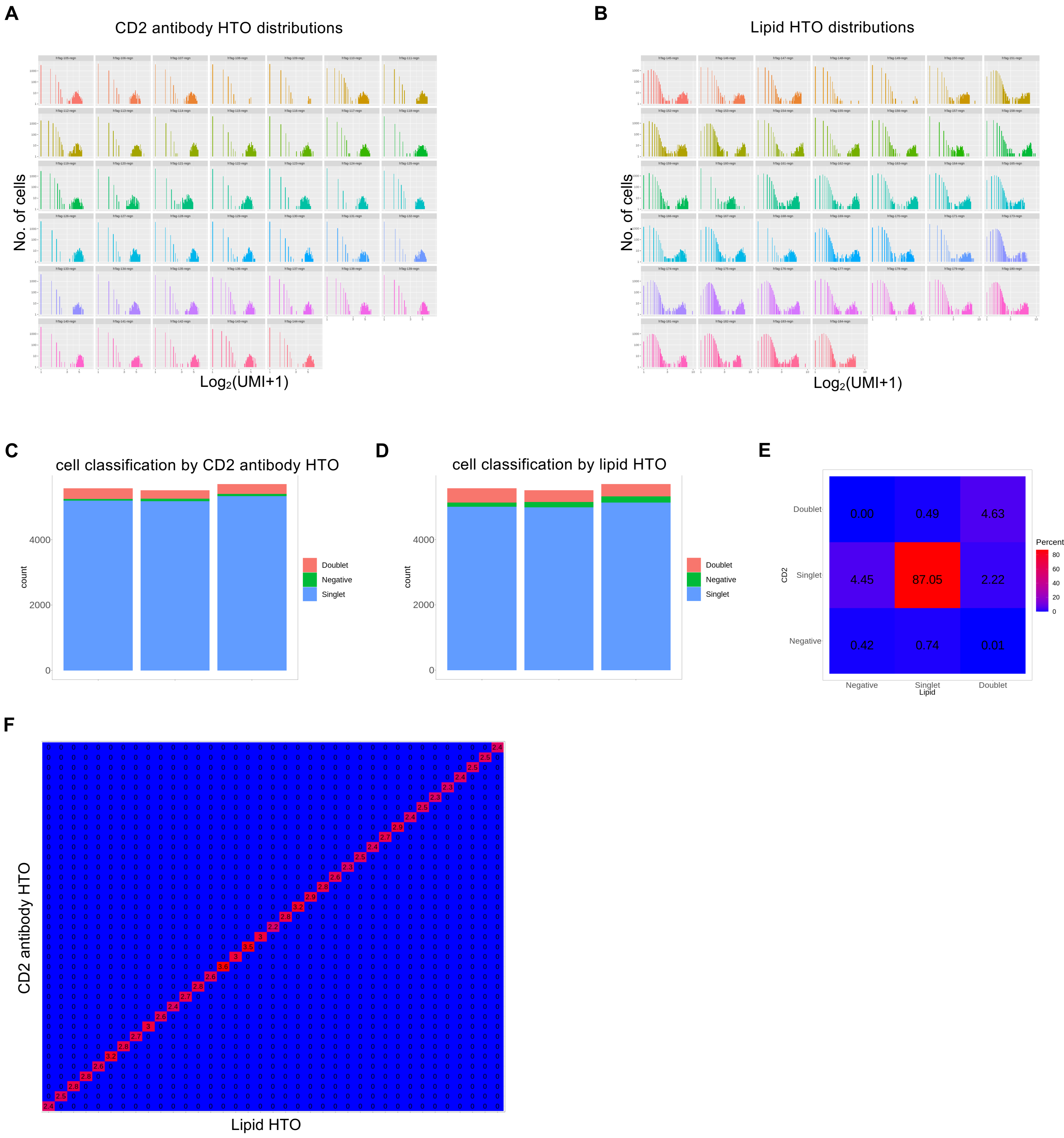


**Supplementary Figure 1: Both anti-CD2 antibody- and lipid-based hashing reagents enable efficient identification of T cells based on HTO classification.** Each aliquot of T cells is labeled with two HTOs; a uniquely barcoded anti-CD2 antibody and a separate, uniquely barcoded lipid HTO. After staining, the aliquots are washed and pooled for capture. (**A** and **B**) Histograms of raw HTO UMI distributions for each anti-CD2 antibody (**A**) or lipid HTO (**B**). (**C** and **D**) Number of cells identified as doublet, negative, or singlet based on independent classification using anti-CD2 antibody (**C**) or lipid HTO (**D**) demultiplexing. Triplicate captures are shown. (**E**) Comparison of cell classification between anti-CD2 antibody and lipid HTOs. Lipid HTO demultiplexing is largely concordant with anti-CD2 antibody HTO classification. (**F**) Matrix showing the percentage of total cells classified by all possible antibody:lipid HTO pairs. The input pairing for each T cell aliquot is on the diagonal and is recovered by HTO demultiplexing.

# Supplementary Figure 2

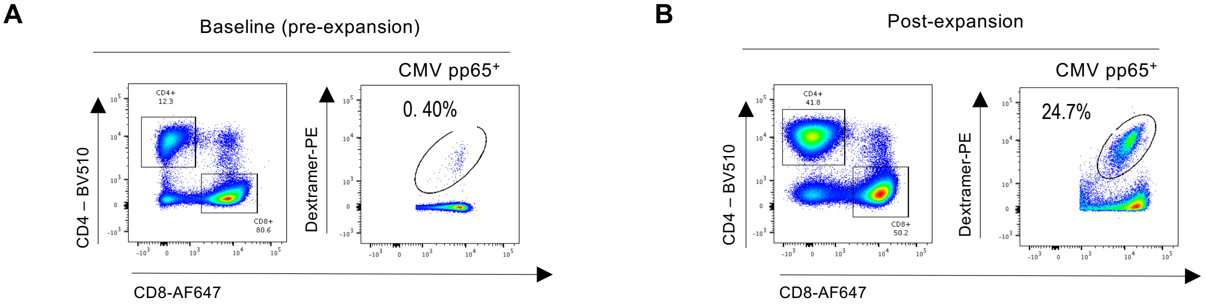


**Supplementary Figure 2: CMV pp65$^+$ T cells were expanded in culture to enable proof-of-concept experiments. Related to Figure 2.** (**A**) The baseline percentage of CMV pp65 dextramer$^+$ CD8$^+$ T cells from a CMV serum positive, HLA-A*02:01$^+$ healthy donor (HD1) was assessed by flow cytometry.
(**B**) HD1 T cells were expanded in culture for 10 days in the presence of CMV pp65 peptide (NLVPMVATV) and DC and T cell supporting cytokines. Post-expansion, the percentage of CMV pp65 dextramer$^+$ CD8$^+$ T cells was determined by flow cytometry.

# Supplementary Figure 3

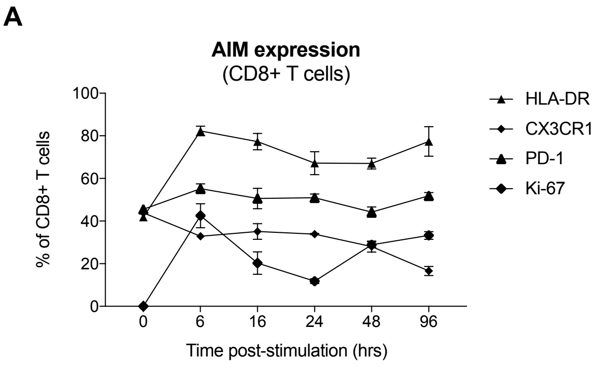


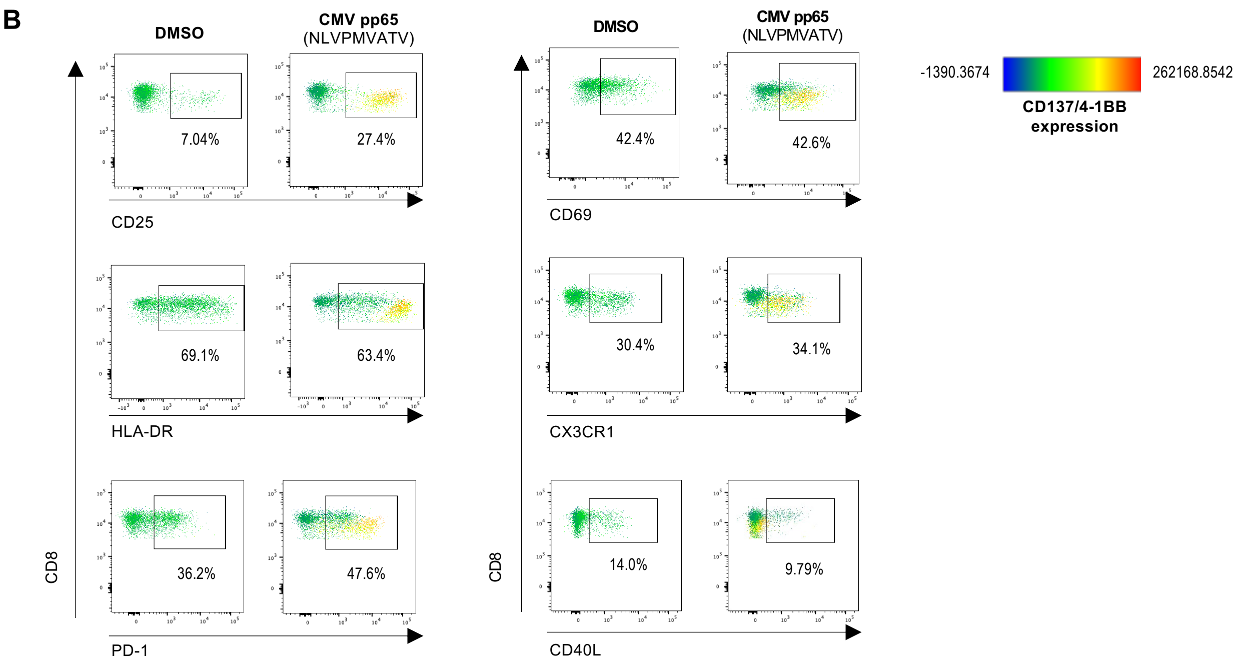


**Supplementary Figure 3: Activation-induced markers are upregulated on antigen-specific T cells following antigen-specific activation. Related to Figure 2.** T cells from a CMV serum positive, HLA-A*02:01$^+$ healthy donor (HD1) were expanded for 10 days in the presence of CMV pp65 peptide (NLVPMVATV) to enable proof-of-concept tests. (**A**) Expanded T cells were restimulated with cognate CMV pp65 peptide in RAPTER assay culture conditions over a 96-hour time course. The percentages of AIM$^+$ CD8$^+$ T cells were assessed by flow cytometry. (**B**) Expanded T cells were restimulated with DMSO or CMV pp65 peptide (NLVPMVATV) for 24 hrs. The percentages of CD8$^+$ T cells that stain positive for the indicated AIMs were assessed by flow cytometry. The CD137/4-1BB$^+$ CD8$^+$ T cells within each AIM$^+$ gate are indicated by the yellow-red color scale.

# Supplementary Figure 4

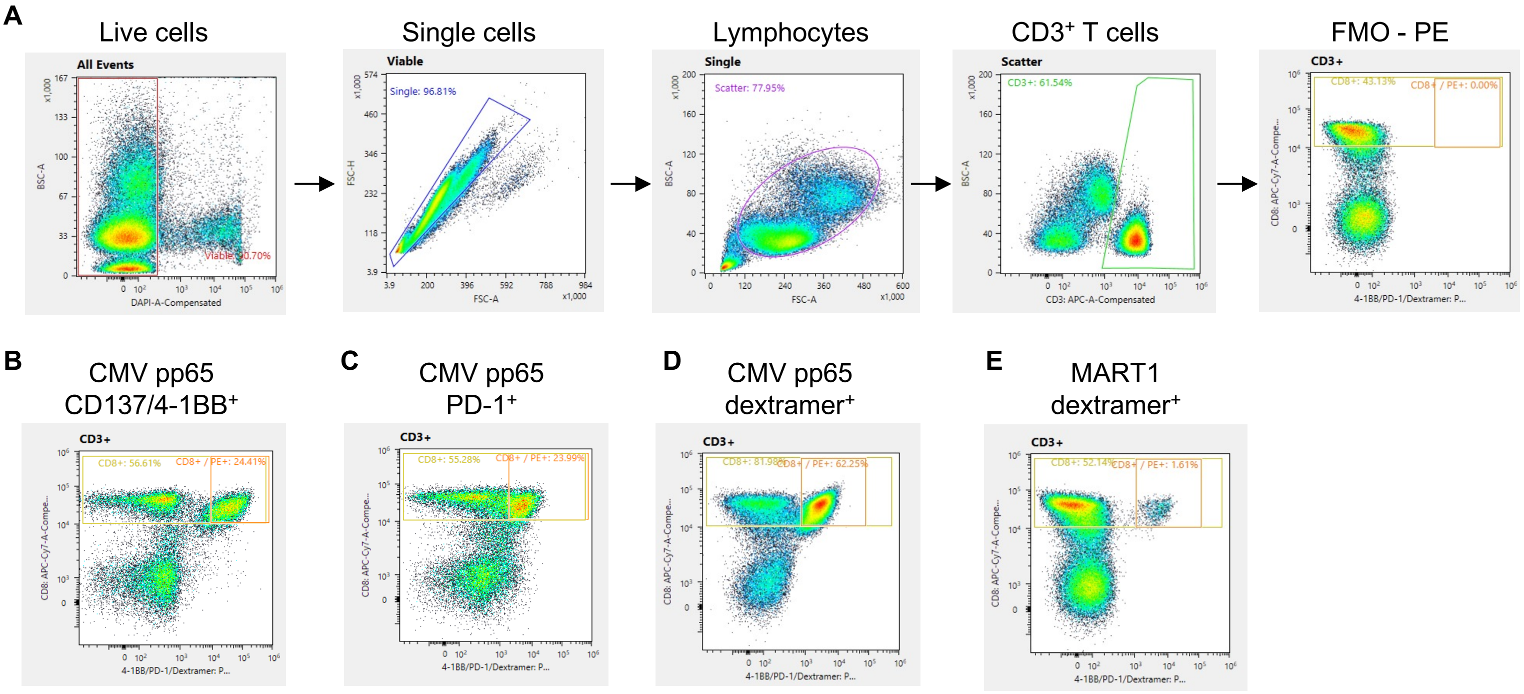


**Supplementary Figure 4**: **Fluorescence activated cell sorting (FACS) gating strategy to isolate CD8$^{+}$ T cell populations for scRNA- and TCR-seq analysis. Related to Figure 2.** (**A**) FACS upper-level gating including the fluorescence minus one (FMO) gate for the PE channel. (**B**) CD137/4-1BB$^{+}$ CD8$^{+}$ T cells. (**C**) PD-1$^{+}$ CD8$^{+}$ T cells. (**D**) CMV pp65 dextramer$^{+}$ CD8$^{+}$ T cells. (**E**) MART1 dextramer$^{+}$ CD8$^{+}$ T cells.

# Supplementary Figure 5

**A**

**13, 241 total captures**

| | Sample | Captures |
|---|---|---|
| 1 | CD8+ | 3748 |
| 2 | CD8+ Mart1+ Dextramer+ | 1527 |
| 3 | CD8+ CMV pp65+ Dextramer+ | 1856 |
| 4 | CMV pp65 restimulated CD8+ CD137/4-1BB+ | 3086 |
| 5 | CMV pp65 restimulated CD8+ PD-1+ | 3024 |

**B**

**6,343 captures post-filtering**

| | Sample | Captures |
|---|---|---|
| 1 | CD8+ | 1809 |
| 2 | CD8+ Mart1+ Dextramer+ | 1127 |
| 3 | CD8+ CMV pp65+ Dextramer+ | 845 |
| 4 | CMV pp65 restimulated CD8+ CD137/4-1BB+ | 1313 |
| 5 | CMV pp65 restimulated CD8+ PD-1+ | 1249 |

Filtering → Paired TCR detected, no multi- chain

**C**

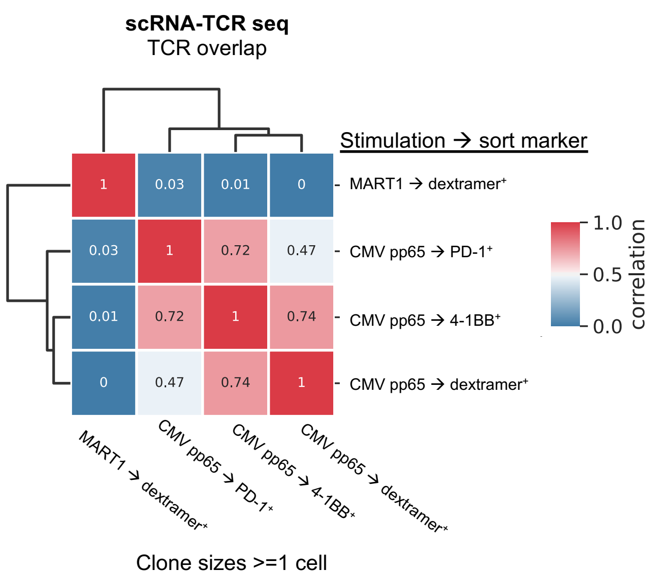


**Supplementary Figure 5: CMV pp65 and MART1 dextramer⁺, and CMV pp65 CD137/4-1BB⁺, and PD-1⁺ CD8⁺ T cells were isolated by FACS then analyzed by scTCR-SEQ. Related to Figure 2.** (**A**) Total scRNA-SEQ captures from all samples pre-filtering and (**B**) post-filtering to select TCR sequences with paired a and b chains. Any TCR captures with multiple chains were eliminated from analysis. (**C**) All dextramer⁺ TCRs were compared to CD137/4-1BB⁺, and PD-1⁺ TCRs with clone sizes >=1 cell.

# Supplementary Figure 6

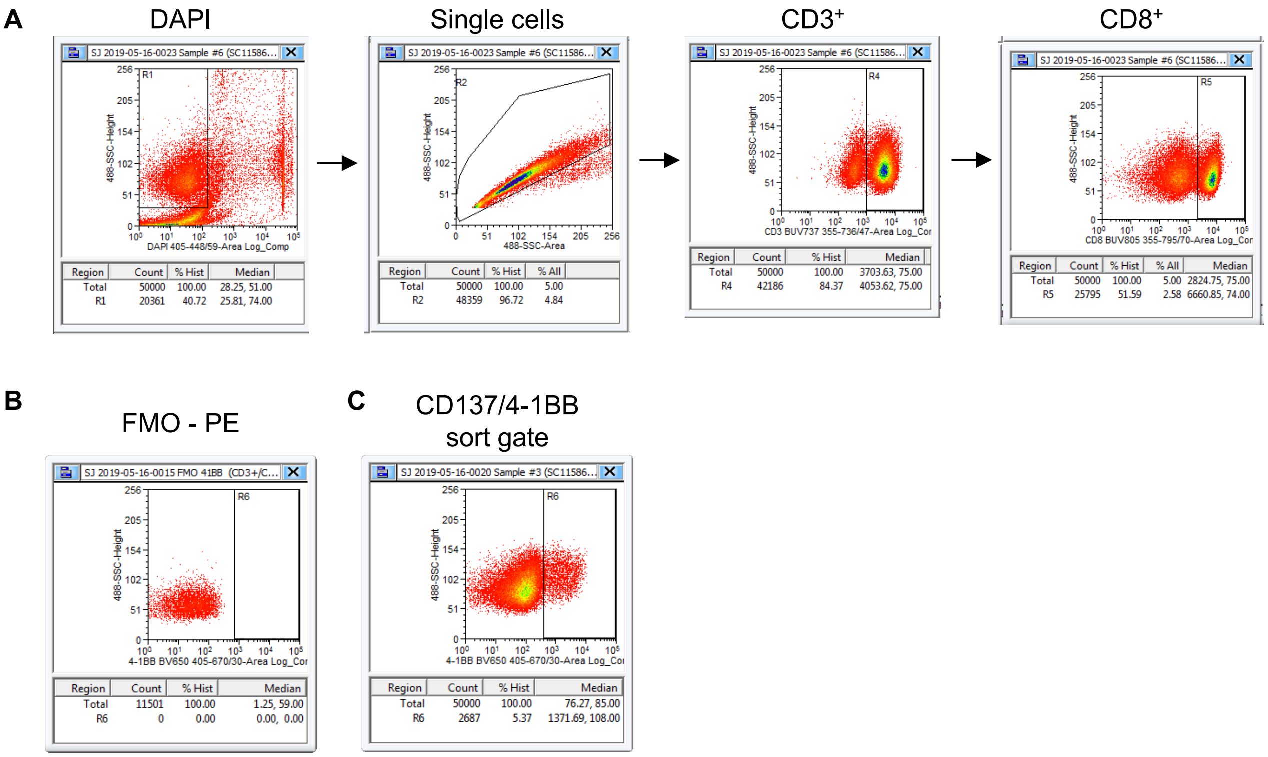


**Supplementary Figure 6: FACS gating strategy to isolate CD8⁺ CD137/4-1BB⁺ T cell populations for scRNA- and TCR-seq**. **Related to Figure 3.** PBMC from HD1 were cultured for 7 days with 5 viral peptides to expand the number of epitope-specific memory T cells. Expanded PBMC were then re-stimulated with DMSO or cognate peptides for 24 hours. (**A**) FACS upper-level gating. (**B**) FMO gate for the CD137/4-1BB (BV650) channel. (**C**) CD137/4-1BB$^{+}$ CD8$^{+}$ T cell sort gate.

# Supplementary Figure 7

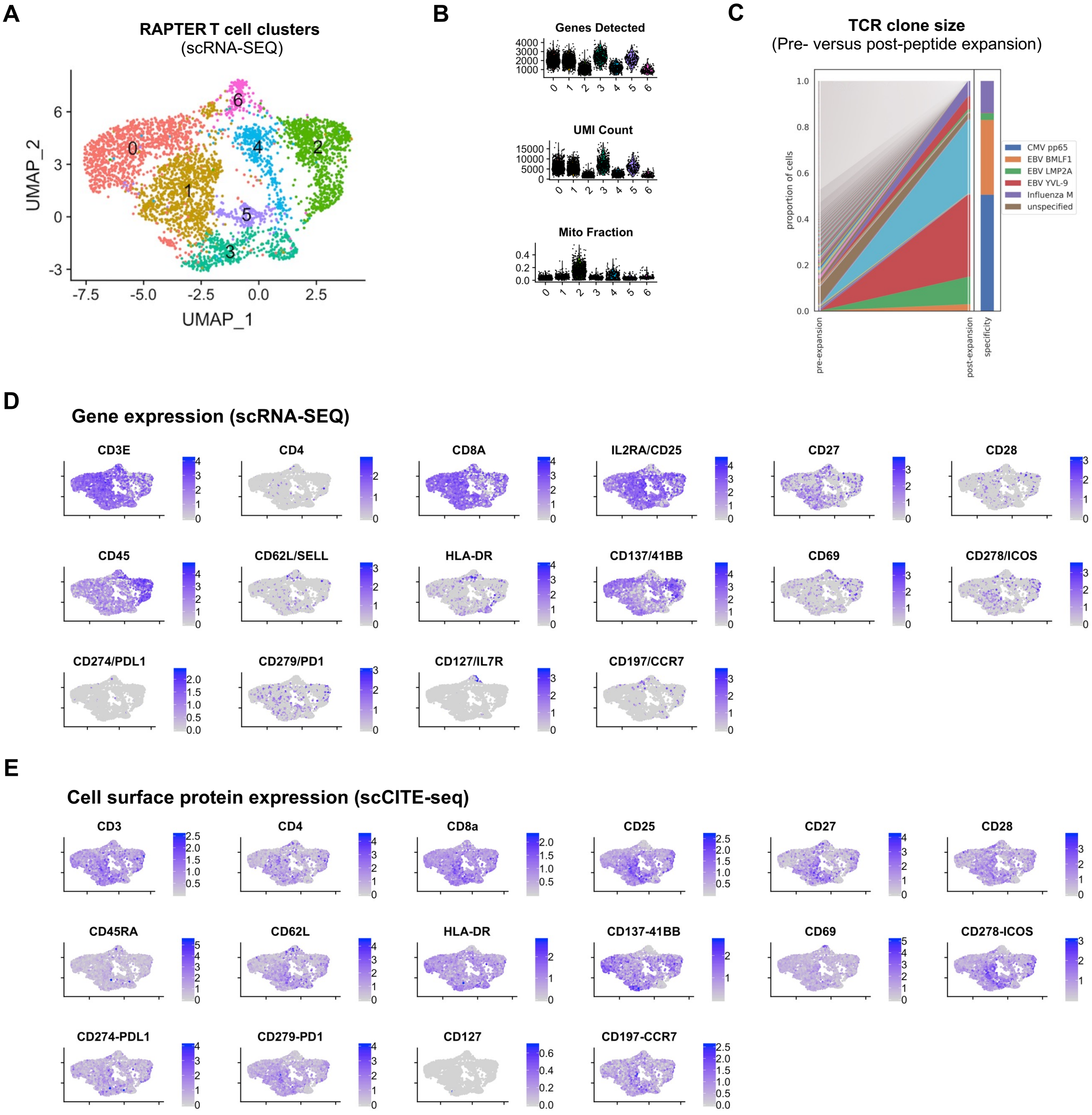


**Supplementary Figure 7: scRNA-SEQ analysis of RAPTER CD8$^+$ T cells. Related to Figure 3.** PBMC from HD1 were cultured for 7 days with 5 viral peptides peptides to expand the number of epitope-specific memory T cells. Expanded PBMC were then re-stimulated with cognate ligand for 24 hours and RAPTER was performed (Figure 3). Unexpanded CD8$^+$ T cells were also analyzed by scRNA- and TCR-SEQ to enable pre- versus post-peptide expansion clone size analyses. (**A**) UMAP of total CD8$^+$ and CD8$^+$ CD137/4-1BB$^+$ T cells enriched by FACS and analyzed by scRNA-SEQ. (**B**) Violin plots for number of genes expressed, unique molecular identifier (UMI) count, and fraction of mitochondrial RNA counts for each cluster. (**C**) Antigen-specific TCR clone size in pre- versus post-peptide expansion samples. (**D**) Feature maps showing selected expression of genes by scRNA-SEQ, and (**E**) scCITE-seq across clusters.

# Supplementary Figure 8

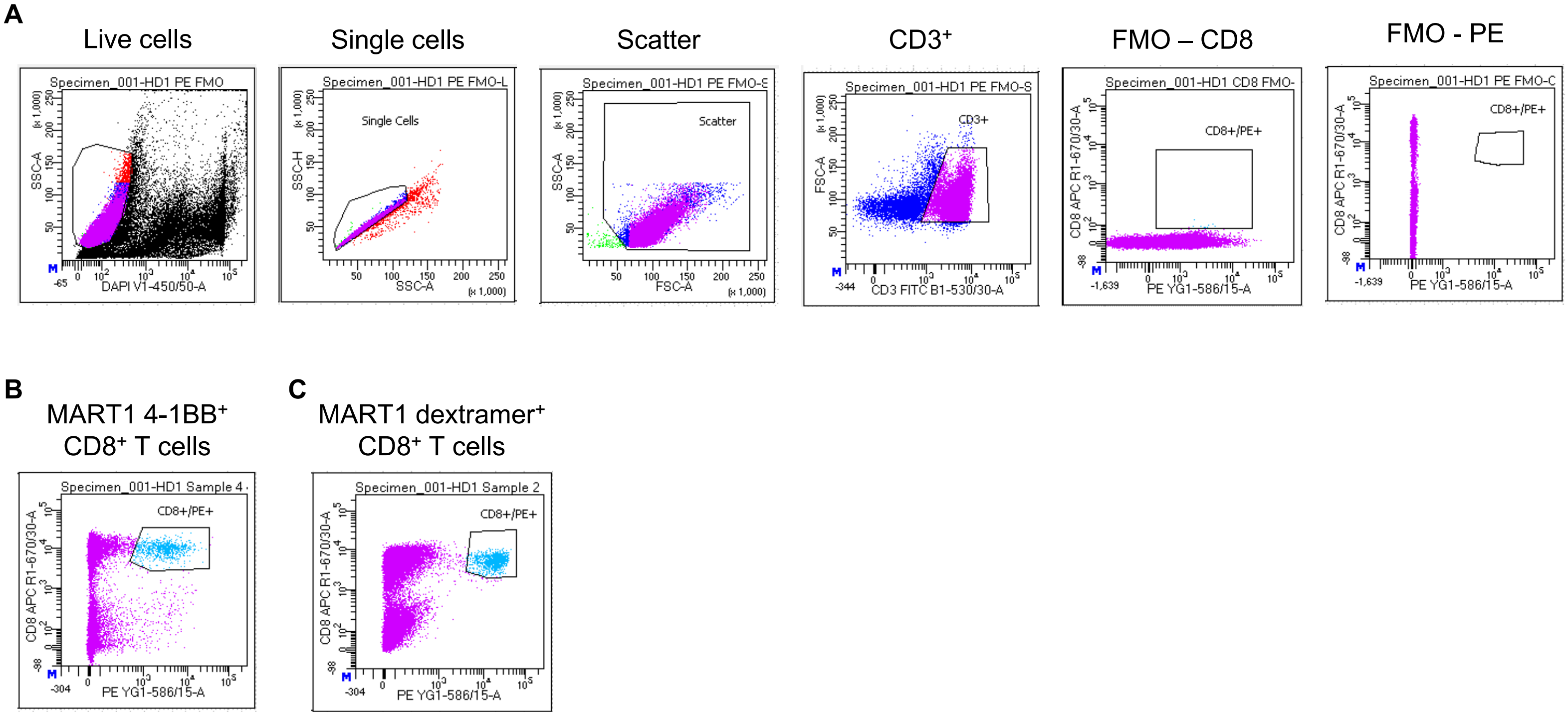


**Supplementary Figure 8: Gating strategies to isolate antigen specific CD8⁺ T cells. Related to Figure 4.** PBMC from an HLA-A*02:01⁺ healthy donor were cultured for 10 days with an HLA-A*02:01-restricted MART1 peptide (ELAGIGILTV) to expand epitope-specific T cells. MART1 peptide-expanded T cells were diluted with unexpanded autologous T cells in a 2-fold dilution series, then re-stimulated for 24 hr with cognate MART1 peptide. Each dilution point was uniquely hashed, then all samples were pooled (Figure 4B). (**A**) Upper-level FACS gating including the FMO gate for the PE channel. (**B**) CD137/4-1BB⁺ CD3⁺ T cells and (**C**) MART1 dextramer⁺ CD8⁺ T cells were isolated by FACS for scRNA/TCR-seq.

# Supplementary Figure 9

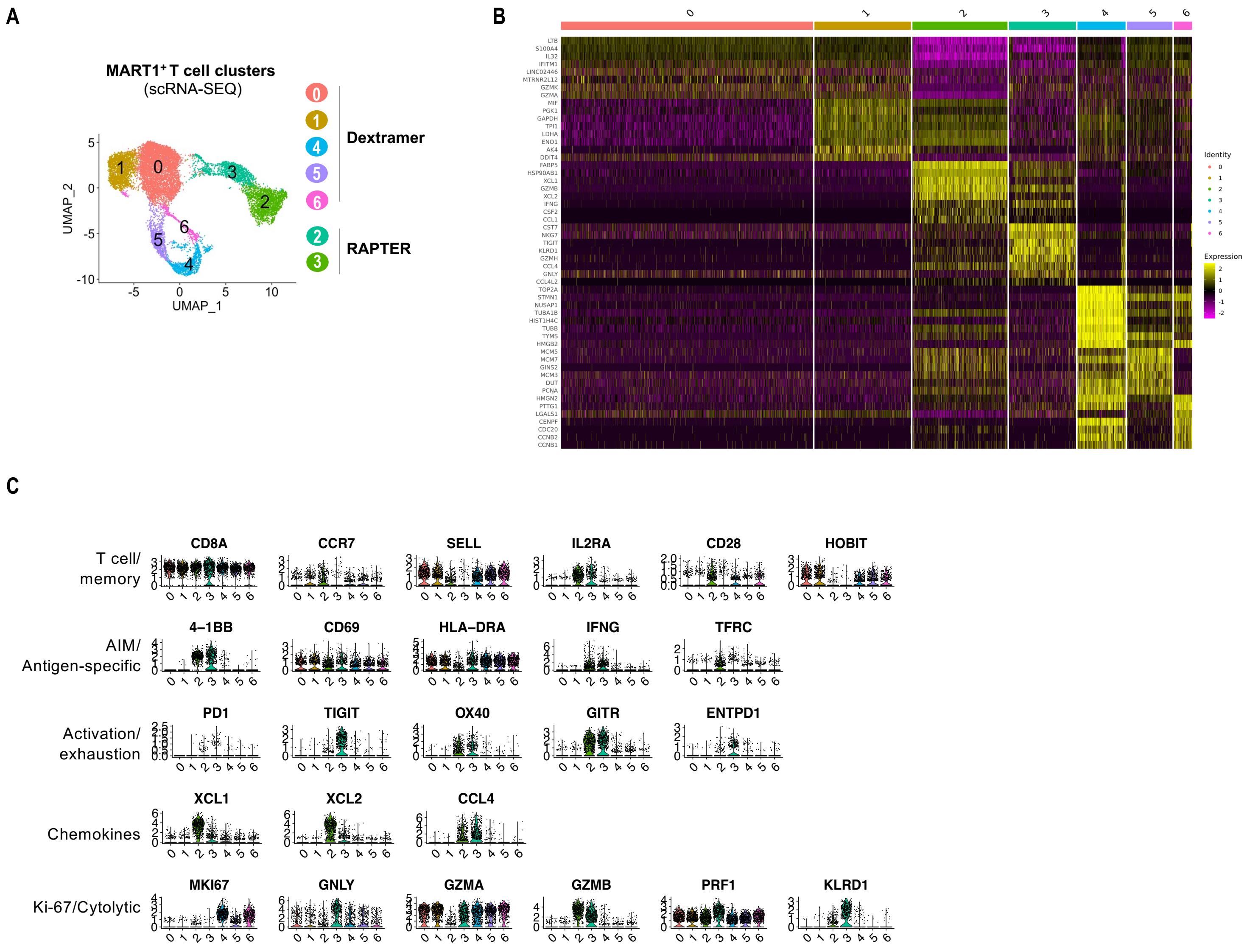


**Supplementary Figure 9: MART1 RAPTER and dextramer sorted CD8⁺ T cell cluster maps and transcriptional phenotypes. Related to Figure 4.** (**A**) Uniform Manifold Approximation and Projection (UMAP) plot of MART1⁺ dextramer-sorted and RAPTER-sorted CD8⁺ T cells. A total of 3,554 CD137/4-1BB⁺, MART1-reactive CD8⁺ T cells were captured and comprised 565 unique clones (red) and 9,930 of MART1 dextramer⁺ T cells were captured and comprised 587 unique clones (aqua). **(B**) Heat map showing the most differentially expressed genes in each cluster. (**C**) Violin plots of relevant phenotypic and functional T cell markers.

# Supplementary Figure 10

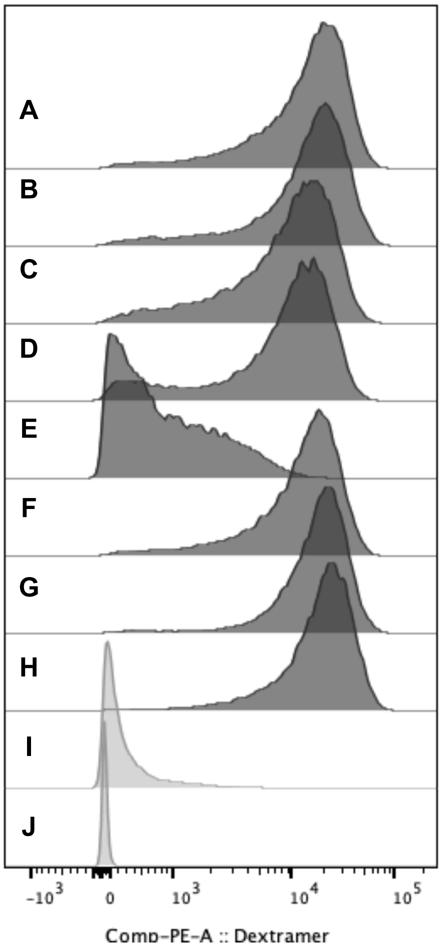


A. MART1 TCR D1
B. MART1 TCR D3
C. MART1 TCR D5
D. MART1 TCR B6
E. MART1 TCR R3
F. MART1 TCR DR3
G. MART1 TCR DR4
H. MART1 TCR DR5

**Supplementary Figure 10. Flow cytometry plots of dextramer specificity of MART1 TCRs identified in the dextramer (A – D), RAPTER (E), or both assays (F – G). Related to Figure 4.** TCR-engineered Jurkat cells expressing the MART1 ELAGIGILTV epitope reactive TCRs were stained with the MART1 ELAGIGILTV dextramer (**A – H**) or a negative control dextramer. Representative negative control dextramer stain (**I**) and FMO control (**J**) are shown.

# Supplementary Figure 11

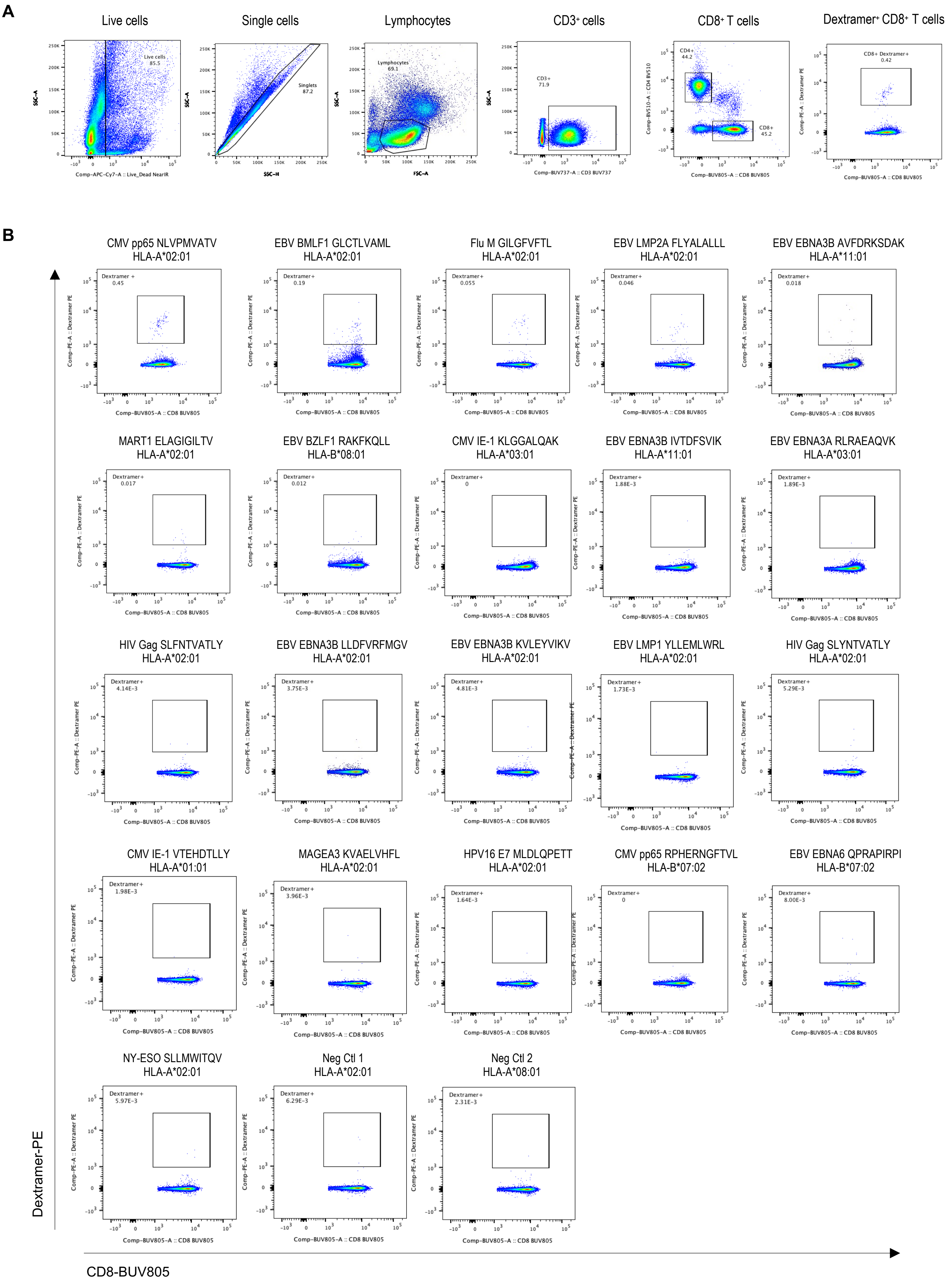

**Supplementary Figure 11: Flow cytometry analyses of frequencies of antigen specific CD8⁺ T cells. Related to Figure 5.** PBMCs from an HLA-A*02:01⁺ healthy donor were stained with individual PE-conjugated oligo-tagged dextramers (HLA haplotype matched and unmatched). Dextramer⁺ cells were gated to assess the abundance of antigen specific CD8⁺ T cells for each epitope. (**A**) Gating strategies for CD8⁺ dextramer⁺ T cells. (**B**). Flow cytometry plots of CD8⁺ dextramer⁺ T cells associated with individual dextramer specificities. The percentage of CD8⁺ T cells that were stained positive for the indicated dextramer is shown in each plot.

# Supplementary Figure 12

**A**

**Pooled dCODE dextramer workflow**

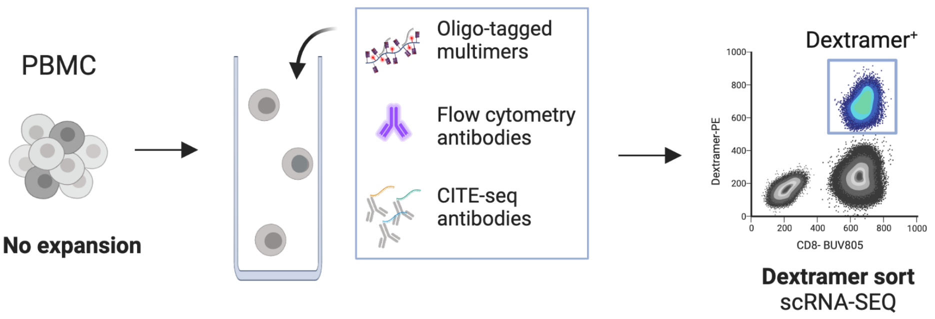


**B**

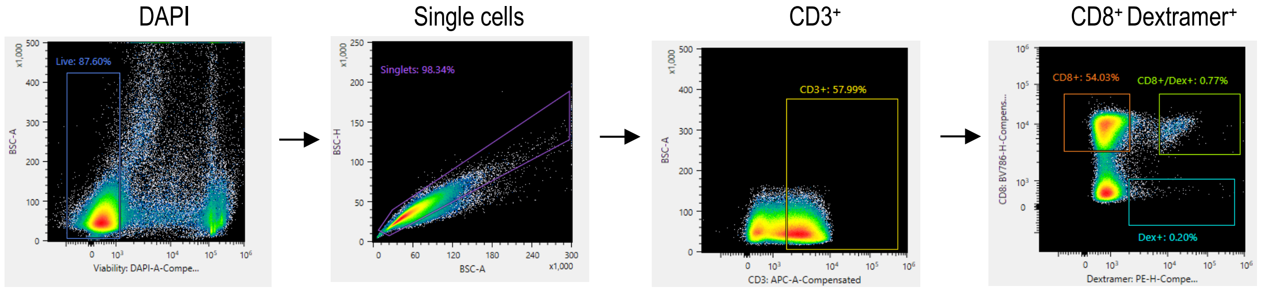


**Supplementary Figure 12: Pooled oligo-tagged dCODE dextramer screen. Related to Figure 5.** (**A**) Schematic summarizing the pooled oligo-tagged dextramer staining workflow. PBMC from a healthy donor were stained with a pool of 23 PE-conjugated oligo-tagged dCODE dextramers (Supplementary Table 1) to detect antigen-specific T cells. Additional flow cytometry and CITE-seq reagents to enable FACS and cell surface protein detection by CITE-seq were also applied. (**B**) Cells were sorted on a Sony MA900 FACS machine to isolate CD8$^{+}$ dextramer$^{+}$ T cells for scRNA-SEQ.

# Supplementary Figure 13

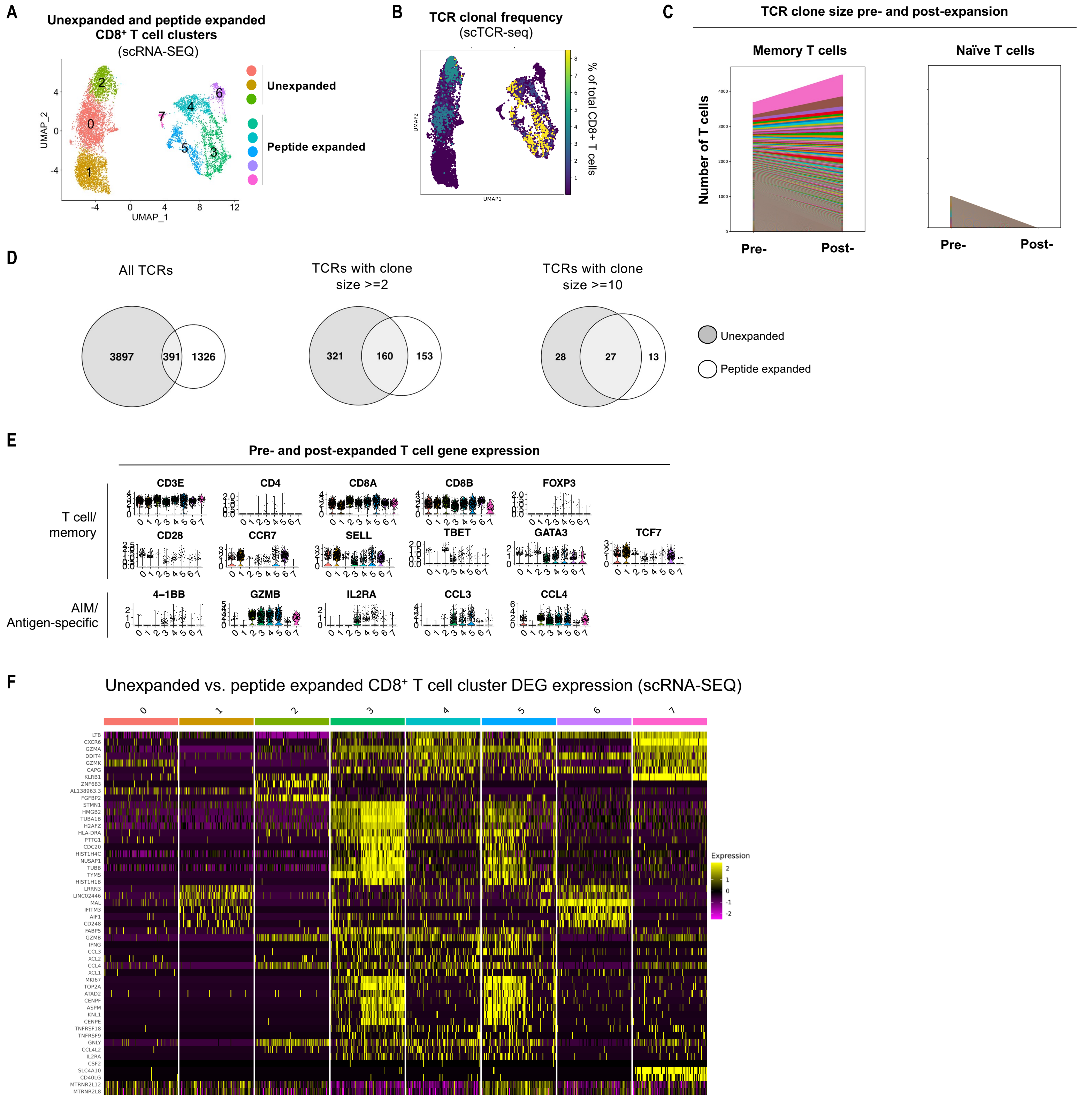


**Supplementary Figure 13: Epitope-specific memory T cell expansion maintains clonal T cells. Related to Supplementary Methods.** PBMC from an HLA-A*02:01⁺, A*29:02⁺, B*35:01⁺, and B*57:01⁺ healthy donor were expanded for 7 days with HLA haplotyped matched and unmatched peptides (Supplementary Table 2) to expand epitope-specific T cells. (**A**) UMAP plot of unexpanded (n=6592 cells) and peptide expanded (n=3453 cells) total CD8⁺ T cells resolved into 7 distinct UMAP clusters using scRNA-SEQ data. (**B**) Feature maps representing the clonal frequencies of total unexpanded and peptide expanded TCR sequences for paired TCR a/b chains. (**C**) Stacked plots representing individual paired TCR a/b chain sequences in the pre-expansion versus peptide expanded CD8⁺ T cells from the memory (left panel) and naïve (right panel) T cell compartments. T cell memory phenotype was determined using RNA- and CITE-seq data. (**D**) Venn diagrams representing paired TCR a/b chain sequence overlap between pre- (gray) and post-expansion (white) CD8⁺ T cells (All clones, clones >=2 cells, and clones >=10 cells). (**E**) Violin plots of relevant phenotypic and functional T cell markers for the unexpanded and expanded CD8⁺ T cells in (A). (**F**) Heat map showing the most differentially expressed genes in unexpanded versus peptide expanded T cell clusters.

# Supplementary Figure 14

A

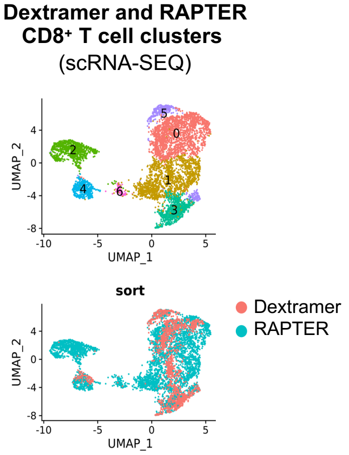


B

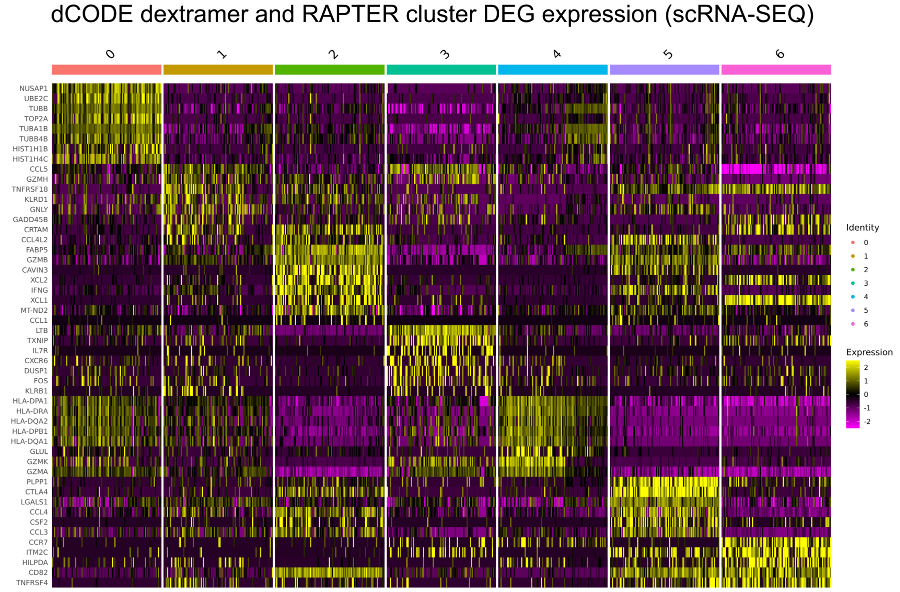


C

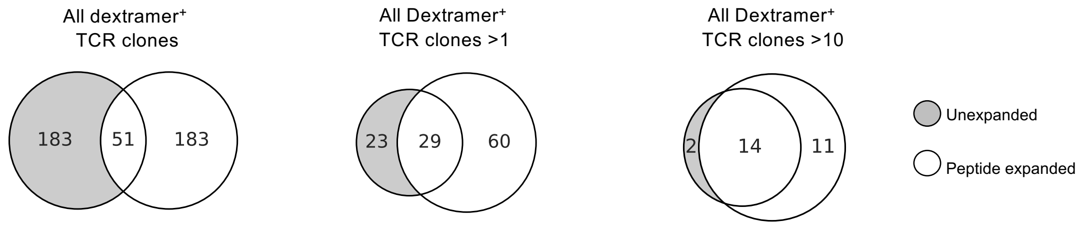


**Supplementary Figure 14: Peptide expanded dCODE dextramer and RAPTER$^+$ CD8$^+$ T cell differential gene expression (DEG) analysis from scRNA-SEQ data. Related to Figure 5.** Total CD8$^+$ T cells from an HLA-A*02:01$^+$ healthy donor were directly isolated by FACS and evaluated by scRNA-SEQ or expanded for 7 days with specific peptides and then isolated by FACS and evaluated by scRNA-SEQ. (**A**) UMAP displaying CD8+ T cell clusters from the dCODE dextramer and RAPTER assays. (**B**) Heat map showing the most differentially expressed genes in the dCODE dextramer and RAPTER$^+$ T cell clusters. (**C**) Venn diagrams representing paired TCR a/b chain sequence overlap between dextramer$^+$ CD8+ T cells pre- (gray) and post-expansion (white) for all TCR clones (left), clone sizes >1 (middle) and >10 (right).

# Supplementary Figure 15

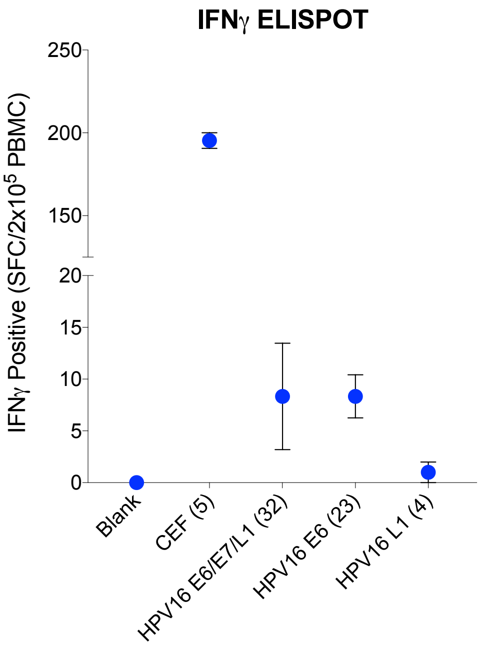


**Supplementary Figure 15: HPV antigen-specific T cell reactivity in PBMC from an HPV$^+$ cervical cancer patient. Related to Figure 6.** Cryopreserved PBMC from an HLA-A*02:01$^+$ cervical cancer patient were tested in an IFNγ ELISpot assay after resting overnight. The number of IFNγ$^+$ spots per $2x10^5$ PBMC indicated was obtained following an overnight stimulation with pools of 15-mer overlapping peptides derived from the HPV proteins E6, E7 and L1, or with short peptides of common viral antigens of CMV, EBV and influenza M (CEF).

# Supplementary Figure 16

**A**

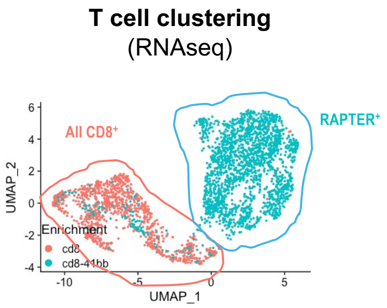


**B**

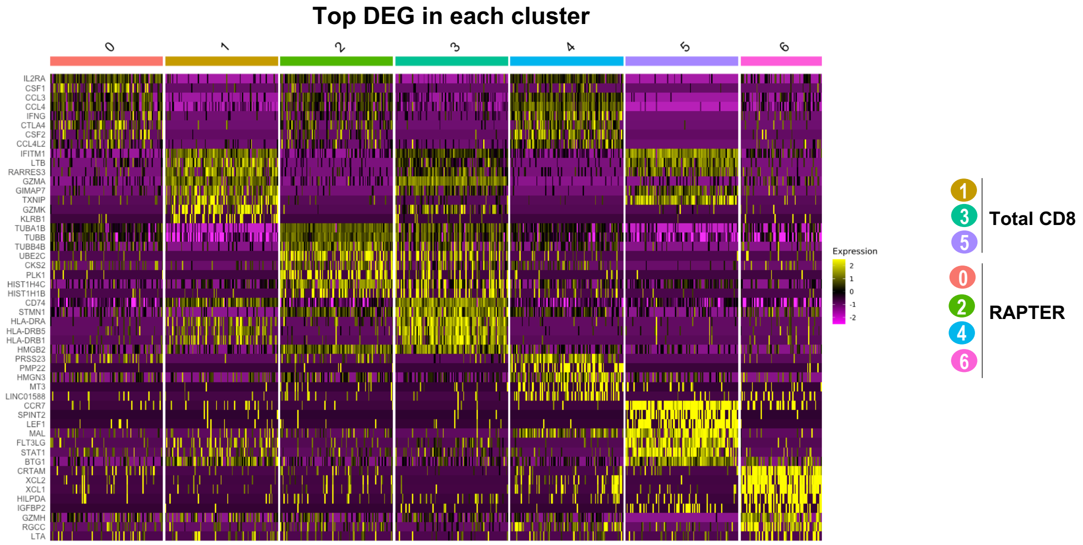


**C**

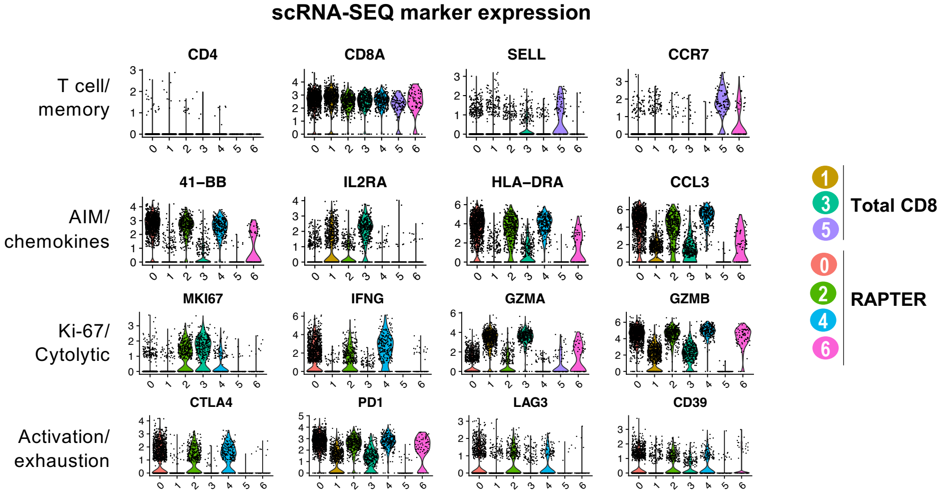


**Supplementary Figure 16: Differential gene expression (DEG) of total CD8+ T cell and RAPTER+ clusters. Related to Figure 6.** (**A**) UMAP of total sorted CD8+ T cells and CD137/4-1BB+ T cells from the RAPTER assay in Figure 6. (**B**) Heat map showing to 10 differentially expressed genes for each T cell cluster in (A). (**C**) Violin plots of relevant phenotypic and functional T cell markers.

# Supplementary Figure 17

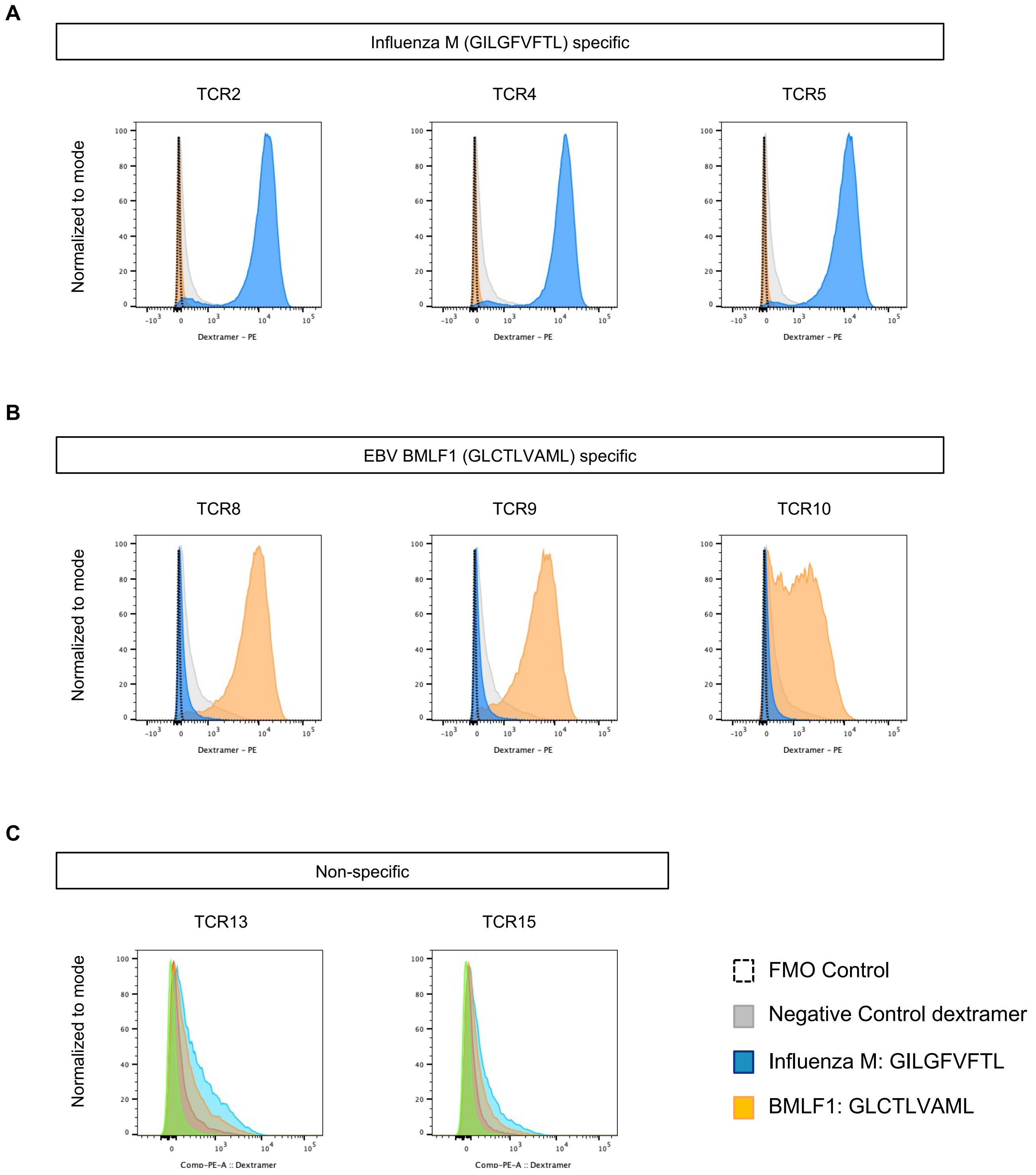


**Supplementary Figure 17. Flow cytometry plots of dextramer specificity of TCRs identified in RAPTER assay. Related to Figure 7.** TCR-engineered Jurkat cells expressing the (**A**) Influenza M (GILGFVFTL) epitope-reactive TCRs (TCR2, TCR4 and TCR6), (**B**) EBV BMLF1 (GLCTLVAML) epitope-reactive TCRs (TCR8, TCR9 and TCR10) or (**C**) two non-specific TCRs (TCR13 and TCR15) were stained with the indicated dextramers.
